# Large-Scale Conformational Analysis Explains G‑Quadruplex Topological Landscape

**DOI:** 10.1021/acs.jpcb.5c04372

**Published:** 2025-09-11

**Authors:** Michał Jurkowski, Mateusz Kogut, Michał Olewniczak, Jan Glinko, Jacek Czub

**Affiliations:** † Department of Physical Chemistry, Gdańsk University of Technology, Narutowicza St 11/12, Gdańsk 80-233, Poland; ‡ Department of Decision Systems and Robotics, Gdańsk University of Technology, Narutowicza St 11/12, Gdańsk 80-233, Poland; § BioTechMed Center, Gdańsk University of Technology, Narutowicza St 11/12, Gdańsk 80-233, Poland

## Abstract

G-quadruplexes (G4) are four-stranded nucleic acid structures formed within sequences containing repeated guanine tracts separated by intervening loop regions. Abundant in the human genome, they play crucial roles in transcription regulation and genome maintenance. Although theoretically capable to adopt 26 different folding topologies—primarily differing in loop arrangements—only 14 of these have been observed experimentally. This raises fundamental questions about whether the remaining topologies are energetically inaccessible and what molecular factors determine the folding patterns of G-quadruplexes. To address these questions, we systematically explored the conformational space of G-quadruplexes using a set of 128 DNA sequences capable of forming two- and three-tetrad structures with varying loop lengths. We conducted foldability evaluations of nearly 20,000 unique G4 conformations obtained through an in silico folding procedure. Our analysis revealed significant differences in foldability among the 26 theoretical topologies. Crucially, we demonstrated that the presence of long-distance propeller loops in 12 of these topologies imposes strict loop length constraints, hindering their formation, especially in sequences with shorter loops. Additionally, we found that the occurrence of long-distance propeller loops is governed by G4 helicity, resulting in opposite folding preferences in right-handed and left-handed G4s. By providing geometric explanation for G4 folding patterns, our study advances the understanding of the G-quadruplex topological landscape and offers valuable insights for the rational design of G4 structures.

## Graphical abstract

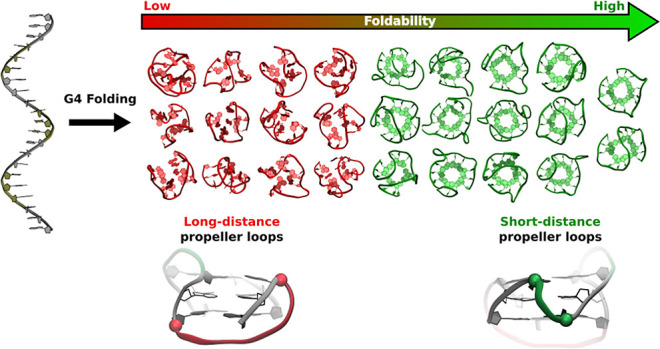

## Introduction

Guanine-rich sequences of nucleic acids have the remarkable ability to fold into noncanonical, four-stranded structures known as G-quadruplexes (G4s).^1–3^ These structures feature a square-shaped core of stacked guanine tetrads (G-tetrads), stabilized by Hoogsteen hydrogen bonds and centrally placed metal ions (see Figure 1A).^4,5^ The core of G4 structures consists of four guanine tracts (G-tracts), which, in unimolecular G-quadruplexes, are linked by three intervening sequences referred to as loops (I, II, and III in Figure 1A).

Sequences conducive to G-quadruplex formation typically follow the pattern G_*n*_L_I_G_*n*_L_II_G_*n*_L_III_G_*n*_, where *G*_*n*_ represents continuous G-tracts of length *n* and L_I–III_ are the loops connecting them. Extensive analysis has shown that the human genome contains over 700,000 potential motifs matching this pattern, which are likely capable of forming G-quadruplex structures.^6^ Among these, around 120,000 have been observed to form stable G-quadruplexes in cells, as demonstrated by a recent genome-wide ChIP-Seq assay.^7^ The same comprehensive study found that G4 structures are prevalent in more than 60% of promoters, especially near transcription start sites, and are present in approximately 70% of genes.^7^

Beyond gene promoters, DNA G4 structures occur in other regulatory genomic regions such as telomeres, immunoglobulin switch regions, and origins of replication where they play pivotal roles in genome maintenance, gene expression regulation, and replication processes.^8–19^

The multiple possible arrangements of DNA strands into compact G4 units leads to intricate folding landscape of G4-forming sequences. This structural diversity arises primarily from different types of connecting loops which also affect the stability and functional versatility of G4s.^20–23^ Depending on the relative orientation of consecutive G-tracts and the direction of strand progression within a G4 structure, five different types of loops can be distinguished (see “Loop types” in Figure 1A). Specifically, propeller loops connect guanines in the bottom and top G-tetrads, spanning between two adjacent G-tracts in either a clockwise (+p) or anticlockwise (−p) direction. In contrast, lateral loops link adjacent G-tracts by connecting guanines within the same G-tetrad, also in two possible directions (+l, −l). Finally, diagonal loops (d) connect guanines from two diagonally opposite G-tracts within the same G-tetrad. Combination of these five loop types, while maintaining strand and G-tracts’ continuity, leads to 26 standard G4 looping topologies^24–26^ (see Figure 1B). Notably, only 14 out of these 26 topologies have been confirmed by high-resolution structural studies so far (indicated by green outlines in Figure 1B and listed in Tables S1 and S2 in Supporting Information). Of these, 13 have right-handed helicity, while only one—the recently reported +p+p+p topology—shows left-handed helicity.^27,28^ It remains unclear why other topologies have not been identified and, more broadly, what the molecular underpinnings of G-quadruplex folding patterns are.

**Figure 1:**
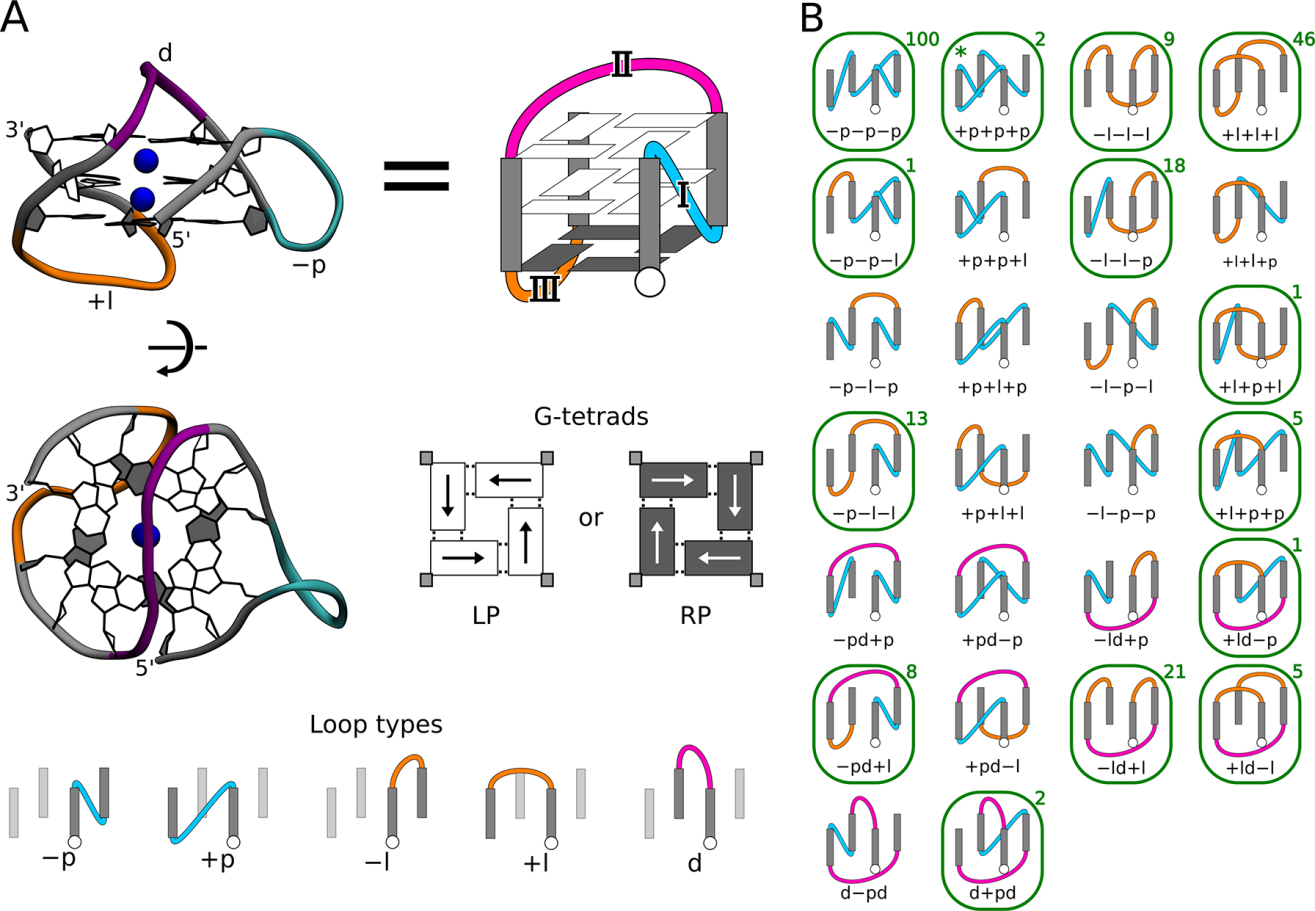
(A) Side and top view of an example G-quadruplex structure featuring three G-tetrads and the propeller “p” (cyan), lateral “l” (orange), and diagonal “d” (magenta) loops, i.e., “–pd+l” topology. The same – pd+l G4 structure is schematically shown on the right, using the convention adopted throughout the article. The panel also shows two possible polarities of G-tetrads defined by the direction of guanine–guanine H-bonds: left-polarity (LP) and right-polarity (RP), and five loop types connecting G-tracts; the “+” and “–” signs denote a clockwise and anticlockwise direction of DNA strand progression, respectively. (B) Depiction of all 26 possible looping topologies of G-quadruplexes. Note that each topology could, in principle, correspond to either a right- or left-handed G4 conformation. Green boxes indicate topologies observed in high-resolution G4 structures, with the number of such structures deposited in Protein Data Bank given in the top right corner. The +p+p+p topology—found only as left-handed G4—is marked with an asterisk. A list of all these experimental G4 structures can be found in Tables S1 and S2 for three- and two-tetrad structures, respectively.

Over the years, numerous studies have shown that the formation of specific loop types and looping topologies strongly depends on the length of loop regions in the G4-forming DNA strand.^29–34^ Based on these findings and the analysis of all experimentally determined high-resolution structures (see Figure S1 in Supporting Information), several broad conclusions about this sequence–structure relationship can be drawn. Specifically, diagonal loops require at least 3 nucleotides (3-nt) to form due to spatial constraints, and lateral loops shorter than 2-nt are extremely rare, with only one solved G4 fold featuring a 1-nt long +l loop.^26^ Conversely, 1-nt intervening sequences tend to form propeller loops, particularly in an anticlockwise direction (−p).^29,34,35^ Structural analyses have also shown that, depending on G-tracts connectivity, propeller loops may adopt two conformations, linking the top and bottom G-tetrads over either a short or longer distance.^35^ Subsequent molecular dynamics simulations of isolated G4 loop models predicted reduced stability for the latter (hereinafter referred to as the long-distance propeller), indicating that it may hinder G4 formation.^35^ However, it remains unclear how exactly these molecular constraints shape the observed G4 folding patterns and to what extent they prevent the formation of certain theoretically possible structures. Therefore, a complete analysis of the G4 conformational landscape is essential to understand these constraints and to uncover the dependencies between sequence and structure that govern G4 formation.

In one such attempt, Fogolari et al. combinatorially assembled nonredundant fragments of loops and G-cores extracted from available G4 structural data, yielding a set of ∼4400 G-quadruplex models.^36^ Among these, they identified only 14 unique looping topologies, including all 13 right-handed topologies confirmed by high-resolution studies. However, since their approach was limited to conformations derived from the experimentally explored regions of G4 conformational space, it failed to produce the left-handed +p+p+p topology which had not yet been identified. For the same reason this approach did not explain the observed folding patterns or address whether the remaining topologies are energetically inaccessible.

Therefore, in this work, we aimed to thoroughly examine the G4 sequence–structure relationship by exhaustively sampling the space of all possible folded G-quadruplex conformations within 26 standard topologies. To this end, we used a specially designed molecular dynamics-based folding procedure to drive formation of all possible G4 conformations for a systematic set of 128 DNA sequences at atomistic resolution, resulting in 19,968 unique structures. Through extensive analysis of the generated conformations, we evaluated the length-dependent impact of loops’ geometry on the G-quadruplex topological landscape. Most importantly, we directly show that the presence of long-distance propeller loops in 12 looping topologies significantly hinder their formation explaining the pattern of experimentally observed folding topologies. We also demonstrate that this pattern governed by the presence of long-distance propellers is helicity-dependent, reversing in left-handed G4s compared to right-handed ones.

## Materials and Methods

### Folding Procedure

A molecular dynamics (MD)-based folding procedure was employed to drive formation of all possible G4 conformations for a systematic collection of 128 DNA oligonucleotides. These oligonucleotides were designed to form either two-tetrad (G_2_T_*i*_G_2_T_*j*_G_2_T_*k*_G_2_ with *i*, *j*, *k* ranging from 1 to 4) or three-tetrad (G_3_T_*i*_G_3_T_*j*_G_3_T_*k*_G_3_) G-quadruplexes. For detailed description of oligonucleotides preparation and MD parameters see “Folding procedure—simulation systems and MD protocol” in Supporting Information Methods.

In this approach, each DNA strand was gradually folded from an unfolded state to the target G4 structure by threading it onto a unique guanine core reference in a G-tract-by-G-tract manner from the 5′-end (see Figure S2 and Movie S1). The folding process involved steered molecular dynamics and, for each conformation, utilized four reference structures for the guanine core, each containing one more G-tract than the previous reference. Derived from experimental data, these guanine core references were processed so each one matched a specific topology and polarity pattern of the G-tetrads (see “Preparation of guanine core references” section in Supporting Information Methods and Figure S3 for details).

The formation of the desired G4 conformation involved four steps, in which a moving harmonic potential with a spring constant of 10,000 kJ/(mol nm^2^) was applied to the RMSD from the reference structure to guide the folding of consecutive G-tracts into their target positions within the guanine core (over 12 ns per step; see Figure S2 and Movie S1). After the complete formation of the intended G4 conformation, to facilitate local relaxation of the twist angle between G-tetrads, we restrained each tetrad separately and continued the simulation for another 12 ns. This was achieved by applying harmonic potential with a spring constant of 5000 kJ/(mol nm^2^) keeping the RMSD from the reference G-tetrads below 0.05 nm.

All generated G4 structures are available at the following DOI: 10.34808/fcyz-w866.

### Validation of G4 Conformations—RMSD Thresholds

To determine whether the structures obtained by our folding procedure reached the intended G4 conformations, we measured the root-mean-square deviation (RMSD) between the guanine core and the unique guanine core reference prepared for each conformation (see “Preparation of guanine core references” in Supporting Information Methods for details). RMSD thresholds, below which structures were considered properly folded, were established based on the guanine core fluctuations in the folded state, separately for three classes of G4 structures: three-tetrad right-handed, two-tetrad right-handed, and two-tetrad left-handed G4s. To determine the ranges of these fluctuations, we conducted unbiased explicit solvent MD simulations initiated from experimental structures belonging to each of the above-mentioned classes. Specifically, we performed three separate 1 μs MD simulations for the three-tetrad right-handed G4s, three 1 μs simulations for the two-tetrad right-handed G4s and one 3 μs simulation for the left-handed G4 (see “Validation of G4 conformations—simulation systems and MD protocols” in Supporting Information Methods and Table S3 for details).

Based on the obtained MD trajectories, we calculated the RMSD values of the G-tetrad stack relative to their corresponding reference structures. This allowed us to generate three separate distributions of RMSD values, characterizing the range of guanine core fluctuations in each of the three considered G4 classes (Figure S4). Subsequently, we defined the RMSD thresholds for each class as 99th percentile of the corresponding distribution. Established threshold values of 0.104, 0.079, and 0.083 nm for three-tetrad right-handed, two-tetrad right-handed, and two-tetrad left-handed G4s, respectively, can be understood as the upper limits of the deviation between the guanine core and the reference in the folded state and thus provide a good distinction between properly and improperly folded structures.

Finally, we labeled all G4 structures obtained by our folding procedure as “foldable” if their RMSD to the unique guanine core reference was below the corresponding threshold value, or as “unfoldable” otherwise.

## Results and Discussion

### Exhaustive Conformational Search Reveals Remarkable Differences in Foldability Between Different G4 Topologies

To comprehensively evaluate the capability of G4 sequences to form various G-quadruplex conformations, we drove the formation of 26 theoretically possible right-handed three-tetrad G4 topologies using a steered-MD-based de novo folding procedure (see Methods and Figure 2A) for the set of 64 DNA oligonucleotides with the general sequence G_3_T_*i*_G_3_T_*j*_G_3_T_*k*_G_3_, where loop lengths *i*, *j*, *k* varied independently from 1 to 4. This exhaustive conformational search encompassed all 8 possible G-core polarity patterns (see Figures 1 and S5), across 26 topologies, resulting in 208 (26 × 8) standard G4 folds per sequence and a total of 13,312 unique three-tetrad G4 conformations, representing the complete G-quadruplex conformational landscape for 64 considered sequences. The same procedure was applied to 64 sequences G_2_T_*i*_G_2_T_*j*_G_2_T_*k*_G_2_ to drive the formation of 6656 two-tetrad folds, thus mapping the G4 conformational landscape for the considered sequences with 2-nt G-tracts (with 4 possible G-core polarity patterns).

**Figure 2:**
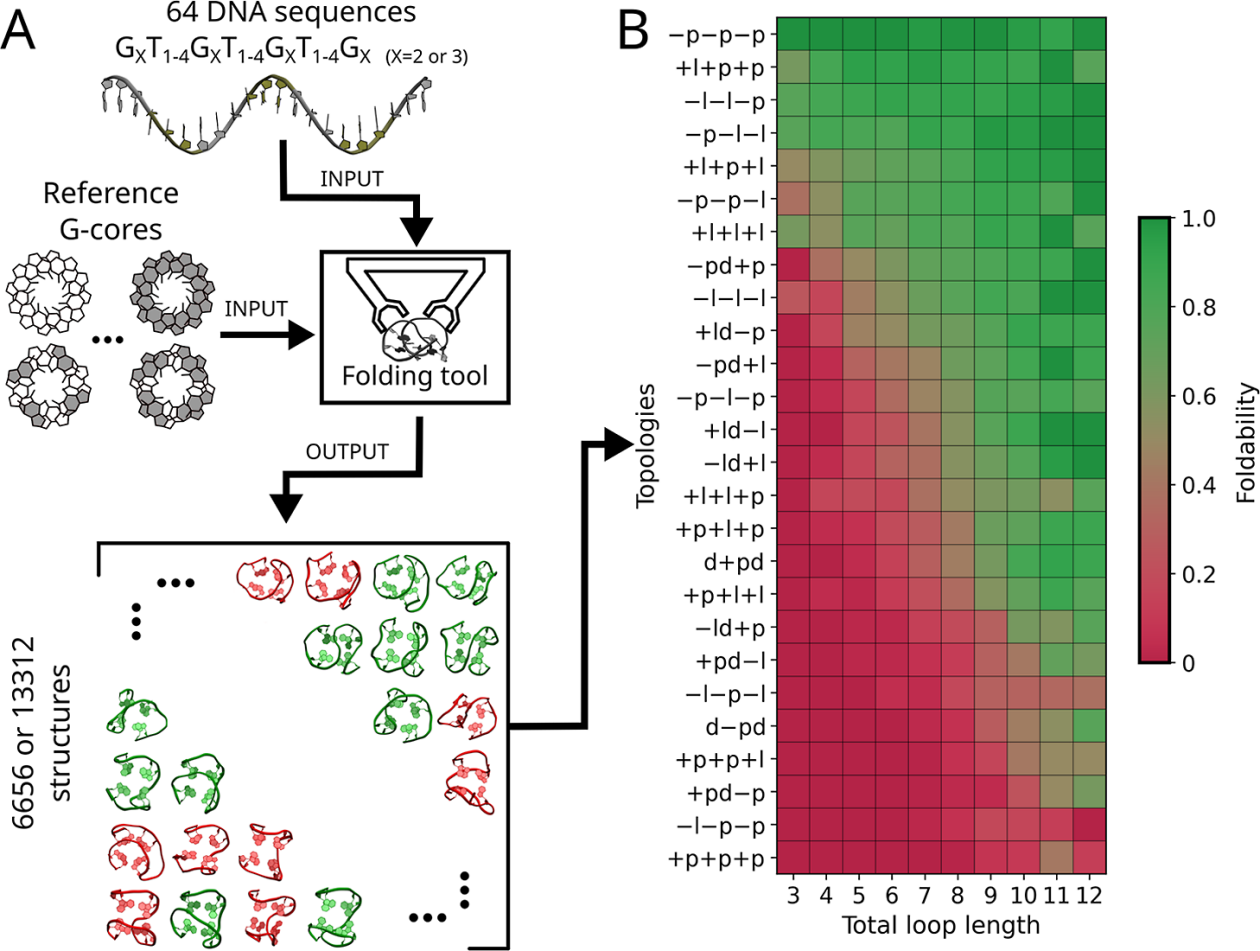
(A) Procedure used to drive the formation of all theoretically possible three- and two-tetrad right-handed G4 folds. A set of 64 DNA oligonucleotides is folded based on the reference G-core structures using steered MD-based de novo folding process (“Folding Tool”; see Methods, Supporting Information Methods and Movie S1 for details). The final outcome of the procedure is a set of all theoretically possible two- and three-tetrad G-quadruplexes, with green-colored structures corresponding to well-folded ones and red-colored structures representing improperly folded configurations. All generated structures are available at the following DOI: 10.34808/fcyz-w866. (B) Foldability for each of the 26 topologies of right-handed three-tetrad G-quadruplexes, calculated as the fraction of G4-foldable structures among all sequences with the same total loop length (*x*-axis) across all eight polarity patterns. The topologies (*y*-axis) are arranged in order of decreasing average foldability. For the foldability data of topologies calculated for specific sequences of three- and two-tetrad G4s, refer to Figures S7 and S8, respectively.

Importantly, within considered sequences we introduced variability only in the loop lengths, not in their nucleobase content (only thymine nucleobases). We suspect that even though different nucleobases in loops can alter the folding kinetics and stability of G4 folds,^34,37,38^ they only slightly contribute to the strong loop-length-dependent geometrical constraints, which are of the main interest of this work. Accordingly, we used thymines in the loop regions as they exhibit the lowest interference with G4 formation.^37–40^

To identify which of the de novo folded structures achieved the intended G4 conformation, we compared their G-cores against reference structures using root-mean-square deviation (RMSD) as a metric. RMSD thresholds were set at 0.1 and 0.08 nm for three- and two-tetrad G4s, respectively, based on the range of G-core fluctuations observed during 3 μs MD simulations of selected experimental G4 structures (see Methods for details). Structures with RMSD values below the threshold were classified as “foldable”, while those above were considered “unfoldable”. As a result, we obtained 6460 out of 13,312 (48.5%) properly folded three-tetrad G4s and 2740 out of 6656 (41.2%) properly folded two-tetrad G4s (see Figure S6). Importantly, all G4 folds with corresponding experimentally determined structures exhibited RMSD values below the threshold (see Tables S1 and S2), confirming the robustness of our method in generating reliable G4 models. Nevertheless, it should be stated that many of the conformations predicted to be foldable may not manifest experimentally, as detecting subtle stability differences between competing G4 folds exceeds the sensitivity of our simple approach.

To explore the accessibility of different G4 topologies depending on sequence length, we calculated the fraction of foldable structures (“foldability”) for each of the 26 topologies across all sequences with the same total loop length (i.e., total number of nucleotides in all three loops). The resulting foldability map, averaged over all G-core polarity patterns (Figure 2B), shows that for nearly all topologies, the fraction of foldable structures increases with total loop length. This trend is consistent with the expectation that longer loop regions are more accommodating of diagonal and lateral loop configurations.

Strikingly, Figure 2B also reveals a wide variation in foldability between G4 topologies: some are highly foldable regardless of loop length, while others remain almost completely unfoldable, even for the longest sequences. In particular, foldability of −p–p–p topology averaged over all sequences is 98%, while for +p+p+p – only 4%. What is even more surprising is that some of the most foldable topologies seem to differ only slightly from those with the lowest foldability. For instance, the two most foldable topologies, −p–p–p and +l+p+p, differ by just one loop type from the two least foldable topologies, −l–p–p and +p+p+p, respectively. This surprising result indicates that the established length requirements for lateral and diagonal loops alone cannot fully account for these foldability differences, suggesting the need for a deeper understanding of how certain loop types are interrelated within G4 topologies.

We observed very similar foldability patterns for two-tetrad G4 conformations, as shown in Figure S9.

Unlike loop length, G-core polarity patterns have only a minor effect on the foldability of different topologies (see Figure S10), even though they are highly correlated with topologies according to experimental studies. For instance, the −p–p–p topology seems to be compatible only with LP/LP/LP polarity pattern, whereas our foldability data indicate that this topology can be formed in combination with all possible polarity patterns. This suggests that different polarity patterns do not impose significant geometrical constraints on the formation of G4 topologies; however, as demonstrated by both experimental and computational work, they can still affect G4 stability through distinct nucleobase stacking interactions,^41–44^ favoring one polarity pattern over the others.

### G-Quadruplex Foldability Strongly Depends on the Position and Directionality of Propeller Loops

To better understand the structural factors that influence the foldability of different G4 topologies, we examined how specific features—such as loop types and lengths at positions I, II, and III, along with tetrad polarity—affect foldability. Using our foldability data, we trained an XGBoost classifier to predict whether certain combinations of G-quadruplex features would result in a foldable G4 structure (model’s precision of 82%, evaluated through 10-fold cross validation). The impact of each feature on G4 foldability was then quantified by determining the average Shapley value for that feature in our model (see Supporting Information Methods).

Figure 3A shows the 15 structural features with the most substantial impact on G4 foldability. The feature with the most favorable effect is the −p loop at position I (I –p), while the +p loop at the same position (I +p) has the strongest unfavorable effect. Remarkably, the impact of −p and +p loops is reversed when they are at position II: −p disfavors foldability, whereas +p favors it. This 2-fold impact of propeller loops on foldability was also observed for two-tetrad G4s (Figure S12). These findings suggest that the influence of propeller loops on G4 foldability is highly context-dependent, relying on their position and the direction of strand progression. The exact molecular mechanisms underlying this dependence will be elucidated in subsequent sections.

**Figure 3:**
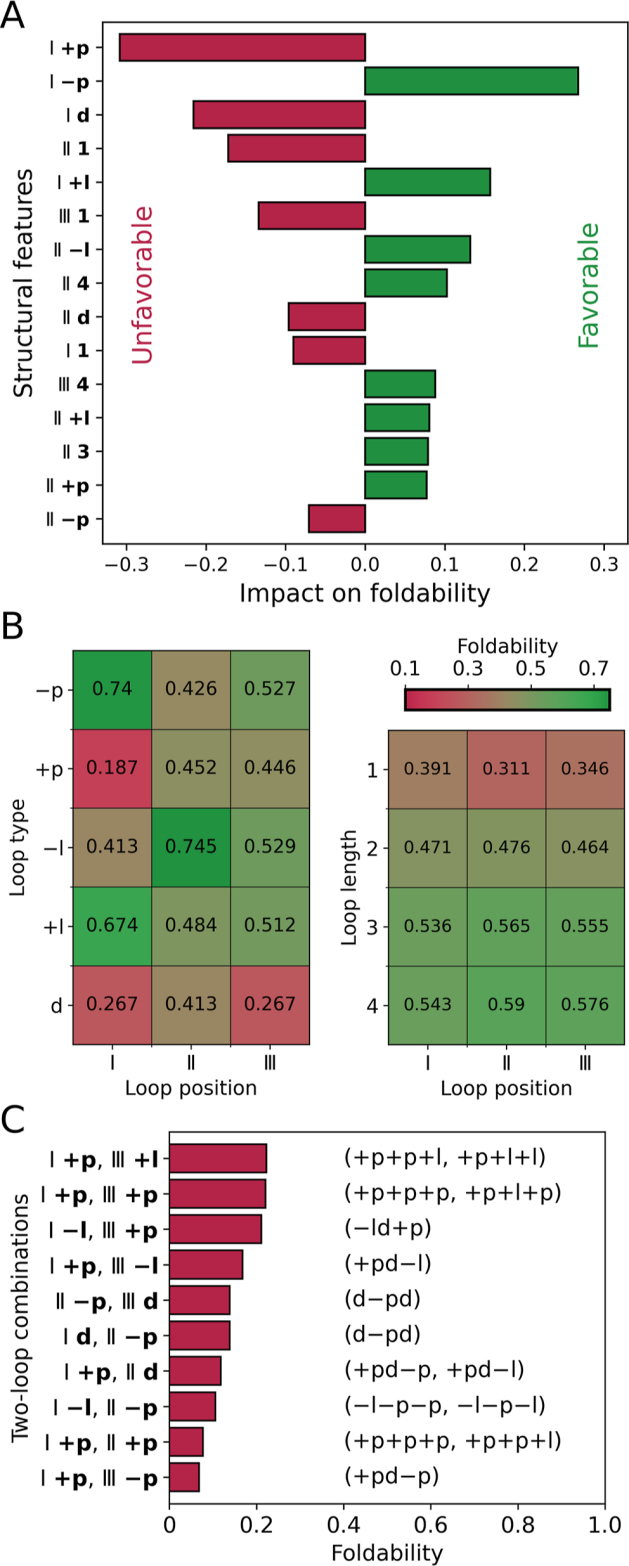
(A) The 15 structural features with the most significant impact on G4 foldability, as determined by their Shapley values in the XGBoost foldability prediction model. Feature labels indicate the loop position (Roman numerals) and the loop characteristic (length or type in bold). This naming convention for G4 structural features is used throughout the paper. For the impact of all considered structural features, see Figure S11. (B) Average foldability of G4 topologies with specific loop types (left) and loop lengths (right) at each of the three positions. Corresponding data for two-tetrad G4s are presented in Figure S13. (C) Average foldability of topologies containing the most unfavorable two-loop combinations (*y*-axis). For the average foldability of all possible two-loop combinations, refer to Figures S14 and S15. The topologies containing each combination are listed on the right. For combinations present in only one topology, the average foldability is simply that topology’s foldability.

Other unfavorable structural features include short 1-nt-long loops (II 1, III 1 and I 1) and the presence of diagonal loops (I d, II d), while the presence of lateral loops (I + l, II –l and II +l) and long 3- or 4-nt-long loops (II 4, III 4 and II 3) clearly favors the foldability.

Therefore, our machine learning analysis not only confirmed the pronounced constraints on the lengths of lateral and diagonal loops but, more importantly, uncovered an unexpected correlation between the directionality and position of propeller loops.

To further explore this correlation, the left panel of Figure 3B compares the average foldability of G4 topologies with specific loop types at each of the three positions. It shows that foldability is highly dependent on the loop’s position. Specifically, G4 topologies with the −p loop at position I are highly foldable (74%), but foldability drops to 43 and 53% when the loop is at positions II or III, respectively. Conversely, the +p loop at positions II and III is associated with much higher foldability (45%) compared to position I (19%), where it is particularly unfavorable. Lateral and diagonal loops also show positional preferences, with −l and d loops being more favorable at position II, and +l loops favoring position I.

In contrast to loop types, no significant dependencies between loop lengths and their positions were observed (Figure 3B, right); foldability simply increases with loop length, in line with our previous observations. Interestingly, length constraints can partially explain the position-dependent differences in foldability for diagonal loops. When a diagonal loop occurs at position I, it also implies its presence at position III, and vice versa (topologies: d–pd and d+pd) This configuration requires sufficiently long loops at both positions, leading to a noticeable reduction in foldability compared to when the diagonal loop is at position II.

### Long-Distance Propeller Loops Greatly Diminish G-Quadruplex Foldability

To understand the molecular mechanism by which the directionality and position of propeller loops affect G4 foldability, we began by examining how the impact of each loop type at a specific position is influenced by the loop types at the other two positions. We calculated the average foldability of topologies with all possible combinations of loop types at any two positions (Figures S14 and S15). Figure 3C shows the most unfavorable two-loop combinations found in topologies with an average foldability of 25% or less. Most of these combinations include the +p loop at position I (I,+p), which our ML model had already identified as a strongly unfavorable feature. However, some combinations, such as I –l/II –p and I d/II –p, include also the −p loop at position II. The average foldability of topologies with these loop pairs (0.1 and 0.15, respectively) is much lower than the average foldability of all topologies containing II –p (∼0.43; see Figure 3B), suggesting that the negative impact of the −p loop at position II is dictated by the preceding lateral or diagonal loop.

In fact, this dual effect of propeller loops on foldability—depending on their directionality and the types of preceding loops—can be explained in simple geometrical terms. To illustrate this, we represented all 26 topologies as schematic two-dimensional projections consisting of G-tracts connected by loops. These projections are obtained by “unwrapping” the three-dimensional representations sequentially by G-tracts, as shown in Figure 4A.

**Figure 4:**
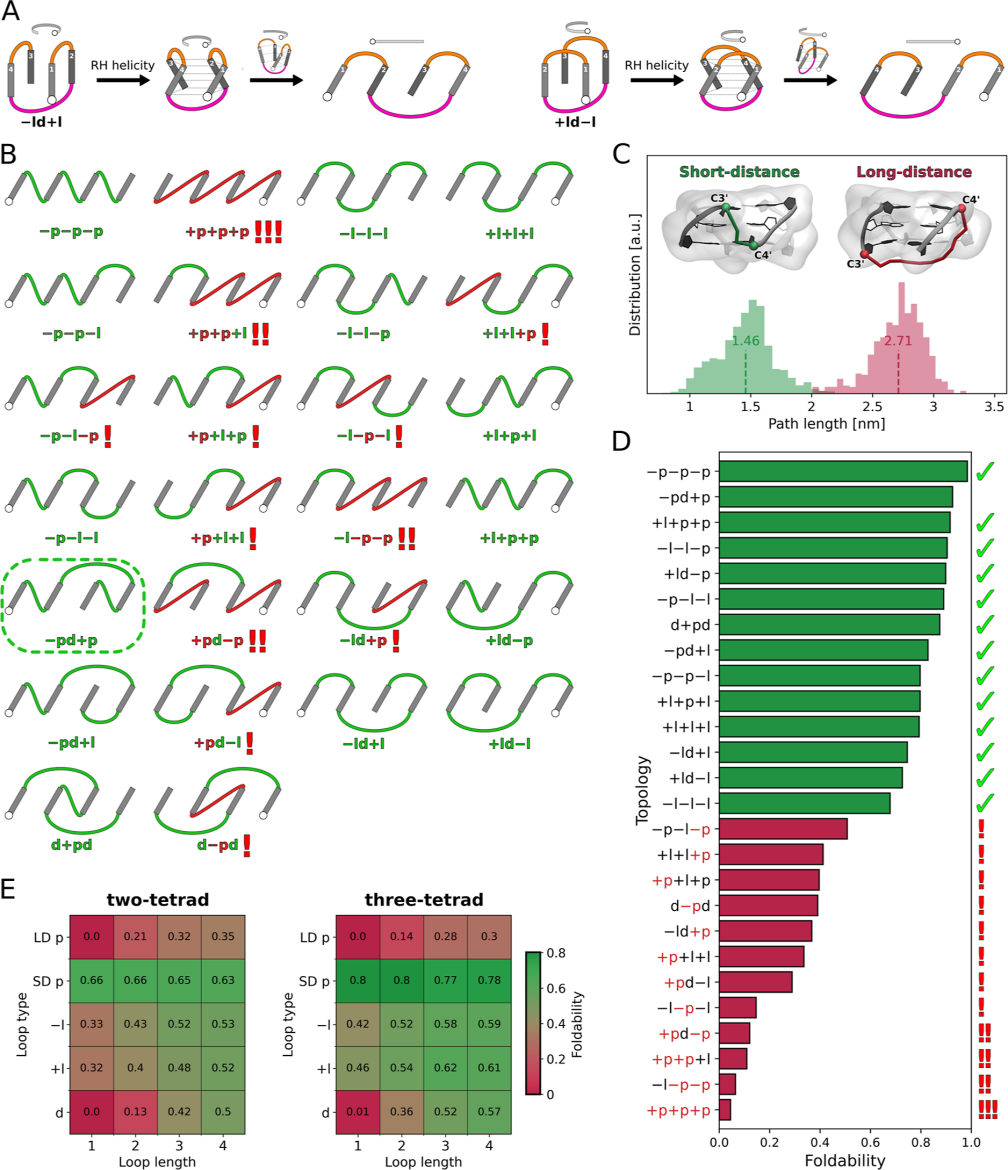
(A) Illustration of obtaining schematic two-dimensional projections of G4 topologies, using −ld+l (left) and +ld–l (right) as examples. After introducing the right tilt of the G-tracts to represent right-handed (RH) helicity–which was omitted in the original representation for simplicity—the topology is sequentially “unwrapped” while maintaining the order of G-tracts (numbered in white). This unwrapping is performed clockwise or counterclockwise for the – and + directionality of the first loop, respectively. As a result, the 5′-end of the G4 (white circle) is located on the left or right side of the 2D projection for the – and + directionality of the first loop, respectively. (B) Two-dimensional projections of all 26 G4 topologies with right-handed helicity. Long-distance propeller loops appear in red, while the other loop types are in green. Exclamation marks indicate the number of long-distance propellers within each topology. The only topology with no long-distance propellers that remains experimentally unsolved, −pd+p, is marked with a dashed green box. (C) Distribution of shortest paths between the attachment points on the connected G-tracts for short- (green) and long-distance propeller loops (red). Vertical dashed lines mark the mean values for the corresponding populations. Examples of shortest paths are shown in the insets for both short- and long-distance propeller loops, with the attachment points (C4′ and C3′ atoms) depicted as spheres. (D) Foldability of each of the 26 right-handed three-tetrad G-quadruplex topologies, calculated across all folded structures while excluding those with diagonal loops shorter than three nucleotides since they are not foldable. Topologies that include long-distance propeller loops are shown in red, with exclamation marks on the right indicating the number of such loops in each topology. Topologies without long-distance propellers are shown in green. Topologies confirmed by experimental right-handed G4 structures are marked with ticks. For the foldability of two-tetrad G4 topologies see Figure S16. (E) Average foldability of G4 topologies with long- (LD) and short-distance (SD) propeller loops, as well as other loop types (−l, +l, d), across all loop lengths ranging from 1 to 4 nucleotides, for both two- and three-tetrad G4 structures with right-handed helicity.

The projections in Figure 4B show two distinct geometries of propeller loops connecting either proximal or distal attachment points of the G-tracts, tilted to the right to represent right-handed helicity. Cang et al. previously identified these as “right” and “left” propeller loops, respectively, noting that the “left” configuration is less stable due to its extended span.^35^ To clarify terminology and avoid overlapping with G4 helicity descriptors, we refer to them here as short-distance and long-distance propellers, respectively.

To compare how different, in terms of the distance spanned by the loop, these two geometries really are, we calculated the shortest path a propeller loop must traverse between the attachment points on the connected G-tracts (see Supporting Information Methods for details) for all properly folded G4 structures in our data set. The path length distribution in Figure 4C displays two distinct populations corresponding to short- and long-distance propeller loops, with mean length of 1.5 and 2.7 nm, respectively, pointing to the significant length constraints for the long-distance geometry.

As can be seen in Figure 4B, 12 out of the 26 looping G4 topologies contain long-distance propeller loops, and so far, none have been observed in high-resolution structures of right-handed G-quadruplexes. This strongly indicates that the presence of long-distance propellers makes the topology energetically much less accessible, in line with previously reported low stability of this loop geometry.^35^ From Figure 4B the general rule emerges for long-distance propellers: the +p loop assumes a long-distance geometry when either no or two nonpropeller loops precede it in the sequence (viewed from the 5′-end), while the −p loop is long-distance when there is exactly one nonpropeller loop before it.

In Figure 4D, we compare the average foldability of all topologies calculated across all potential G4 structures, excluding those with diagonal loops shorter than three nucleotides since they are not foldable. The data reveal that all topologies featuring long-distance propeller loops (red bars) have foldability values below 0.5, which are markedly lower than those of the remaining 14 topologies lacking this structural element (green bars) with foldability ranging from 0.7 to 1.0. This strongly suggests that long-distance propeller loops are a major factor limiting the accessibility of a large portion of the G4 conformational space. Notably, foldability generally decreases as the number of long-distance propellers in a topology increases, with topologies containing three (+p+p+p) or two such loops (−l–p–p, +p+p+l, +pd–p) being the least foldable.

Figure 4D also demonstrates that even though topologies containing a long-distance propeller loop are significantly less foldable compared to those with only short-distance propellers, they still show non-negligible foldability. This indicates that with sufficiently long loop sequences, the long-distance propeller geometry becomes achievable.

To further investigate this, we calculated how the average foldability of topologies containing at least one long-distance propeller depends on the length of this loop. For comparison, the same analysis was conducted for other loop types. Figure 4E reveals that topologies containing 1- and 2-nt long-distance propellers exhibit very low foldability, at 0 and 14% respectively. As predicted, extending the long-distance propeller loops to 3 and 4 nucleotides leads to increased foldability (28 and 30%, respectively); however, these values remain significantly lower than those observed for topologies with diagonal loops of length 3 (52%), which is the minimal length necessary for this loop type in stable G4s. Therefore, these results confirm strong length constraints for long-distance propeller loops and suggest that the minimal length required for this loop geometry in stable G4s is 5 nucleotides or more.

For two-tetrad G4s, topologies with 4-nt long-distance propeller loops also have lower foldability compared to those with 3-nt diagonal loops (35 vs 42%), but the difference is less pronounced than in three-tetrad G4s. This seems to result from less strict length constraints for long-distance propellers in two-tetrad G4s, attributed to the shorter path between their attachment points compared to three-tetrad structures (Figure S17). These results suggest that two-tetrad G4s might more readily fold into topologies with long-distance propeller loops if the loop regions are sufficiently long. Indeed, the NMR structure of the c-kit promoter G-quadruplex with a nonstandard topology (PDB code: 2O3M) features a 3′-end snapback loop that connects two adjacent G-tracts and spans two G-tetrads, adopting a conformation akin to the long-distance propellers seen in two-tetrad G4s (Figure S18).^45^ Notably, this loop comprises five nucleotides, matching the length threshold indicated by our analysis.

### Foldable −pd+p G-Quadruplex—a Candidate for High-Resolution Structure Determination

As depicted in Figure 4D, out of the 14 right-handed topologies lacking long-distance propeller loops only one, −pd+p, has not been reported so far by high-resolution structural studies. Accordingly, we predict that G-quadruplexes adopting this topology are stable and can be obtained through rational design methods, such as sequence adjustments and chemical nucleotide modifications.

Considering the length constraints for lateral and diagonal loops, we propose that an optimal sequence capable of forming this topology should have 1-nt loops at positions I and III and a 3-nt or longer loop at position II. For sequences with this loop length configuration, only two topologies are geometrically feasible: −pd+p and −p–p–p. Notably, several G-quadruplex structures formed by sequences following this pattern have been reported (see Table S1), all adopting the −p–p–p topology. Experimental studies suggest that this topology is often preferred, even if only one or two loops in the sequence are 1-nt-long.^46^ However, this preference could potentially be overridden by substituting 8-bromoguanines in the third G-tract to enforce the guanines’ conformation which sterically hinders the formation of a propeller loop at position II.^44,47–50^ Furthermore, unlike the −p–p–p topology, the −pd+p topology has both the 5′- and 3′-ends located on the same side of the guanine core. This positioning allows for additional stabilization by incorporating 5′- and 3′-flanking sequences capable of forming Watson–Crick pairs.^51,52^

### Right- and Left-Handed G-Quadruplexes Show an opposite Pattern of Foldability

While right-handed G-quadruplexes dominate the experimental high-resolution structures, several left-handed G4s have also been reported.^27,28^ Interestingly, all of them adopt the +p+p+p topology, which, according to our findings (Figure 4D), is the least foldable among the right-handed G4s due to the presence of three long-distance propeller loops. From the schematic projections in Figure 4C, it becomes apparent that whether a propeller loop is short- or long-distance depends on the tilt of the connected G-tracts, indicative of the structure’s helicity (see comparison of left- and right-handed G4 in Figure 5A). Therefore, when the helicity of a G4 with a given topology changes from right- to left-handed, the geometry of propeller loops switches from short- to long-distance and vice versa, as shown by the projections in Figure S19. Given the strong destabilization caused by long-distance propellers, these reversed geometric properties should lead to an opposite foldability pattern between right- and left-handed G4s.

**Figure 5:**
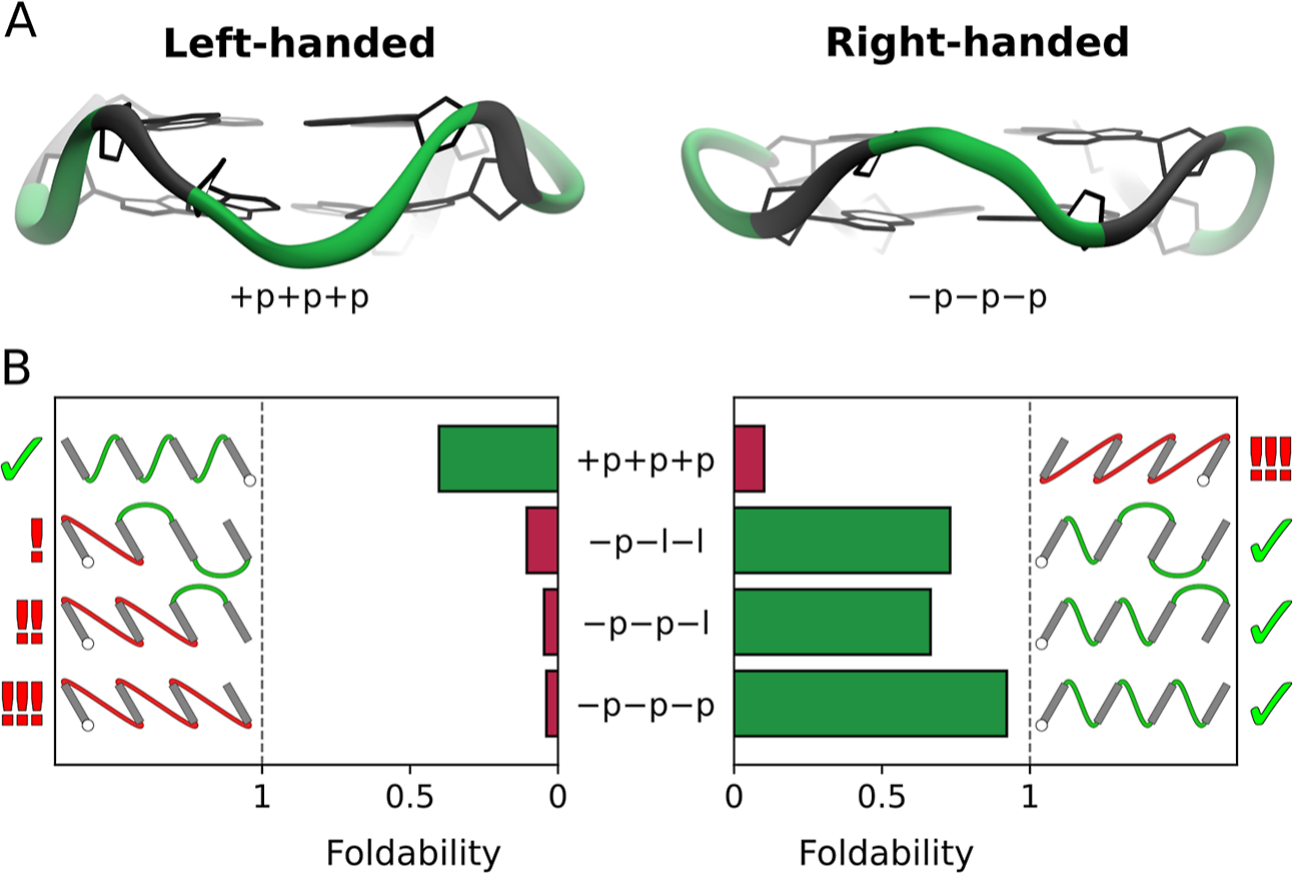
(A) Structures of left- and right-handed two-tetrad G4s with +p+p+p and −p–p–p topology, respectively. Note that G-tracts in these structures (shown in gray) are tilted in the opposite directions, a feature also reflected in the schematic projections of the G4s. (B) Comparison of foldability between left- and right-handed two-tetrad G4s for selected topologies. Two-dimensional projections for each topology with left-handed helicity are shown on the left, and those with right-handed helicity are shown on the right. Topologies that include long-distance propeller loops are shown in red, with exclamation marks indicating the number of such loops in each topology. Topologies without long-distance propellers are shown in green. Topologies confirmed by high-resolution G4 structures for each helicity are marked with ticks.

To test this hypothesis, we applied our folding procedure to induce the formation of left-handed G4s with four different topologies: +p+p+p, −p–l–l, −p–p–l, and −p–p–p, which contain 0, 1, 2, and 3 long-distance propellers, respectively. Consistent with the approach used for right-handed G4s, for each topology, we used the same set of 64 oligonucleotides with the general sequence G_2_T_*i*_G_2_T_*j*_G_2_T_*k*_G_2_, where *i*, *j*, *k* varied independently from 1 to 4, and considered all four possible G-core polarity patterns.

In Figure 5B, we compare the foldability of these four topologies between both helicities. As expected, foldability of +p+p+p is much higher in left-handed G4s than in right-handed ones (40% and 10%, respectively), and it is the highest among the left-handed G4s examined. Indeed, due to the reversed propeller geometry, left-handed +p+p+p G4s are structurally quasi-equivalent to the most foldable right-handed −p–p–p G4s (both containing three short-distance propeller loops) and are thus expected to have the highest foldability among all left-handed G4s. This observation accounts for why +p+p+p is the only topology with left-handed helicity confirmed by high-resolution structural studies to date.^27,28^ Furthermore, Figure 5B demonstrates that foldability of topologies comprising long-distance propellers in left-handed G4s (−p–l–l, −p–p–l and −p–p–p) is very low and decreases with the number of long-distance propellers, similarly to the trend observed in right-handed G4s. This supports the conclusion that the foldability of G-quadruplexes is heavily dependent on long-distance propeller loops.

However, it is important to highlight that the foldability patterns of right- and left-handed G4s are not entirely symmetrical. In fact, left-handed G4s exhibit substantially lower foldability, e.g., the +p+p+p topology is about 2-fold less foldable than its right-handed equivalent, −p–p–p. This difference can be attributed to the inherently higher stability of right-handed G4s, which is thought to result from the more energetically favorable conformation of G-tracts compared to left-handed G4s.^53^

## Conclusions

In this work, we systematically explored the conformational space of standard unimolecular G-quadruplex DNA structures to elucidate the factors shaping their topological landscape. By employing a molecular dynamics-based de novo folding procedure, we systematically generated all theoretically possible three- and two-tetrad G4 folds for a comprehensive set of 128 DNA sequences, yielding over 20,000 unique structures.

Our analysis revealed remarkable differences in foldability among the 26 theoretical G4 topologies.^24^ While some topologies exhibited high foldability regardless of loop length, others remained largely unfoldable even with longer loop sequences. Notably, the directionality and position of propeller loops emerged as crucial factors determining whether a given topology is G4-foldable. This dual effect of propeller loops on foldability is attributed to the existence of two different geometries of these loops: short-distance and long-distance. Long-distance propeller loops, which span a considerably longer path on the G4 structure, impose much stricter loop length constraints and render the topology energetically inaccessible, in line with previous findings.^35^ Our analysis revealed the presence of long-distance propeller geometry in 12 out of 26 topologies among right-handed G-quadruplexes, which explains their substantially lower calculated foldability and accounts for why these theoretically possible topologies have not been observed in high-resolution structural studies. Despite the overall destabilizing effect of long-distance propellers, our search of G4s conformational space also predicts that sufficiently long intervening sequences are capable of adapting this loop geometry, as suggested by high-resolution structural studies.^45^

In contrast, among the right-handed G4s without long-distance propeller loops, only the −pd+p topology has not been observed experimentally. Based on our findings, we predict that G4 structures adopting this topology are stable and could be obtained through targeted sequence design.

Extending our analysis to left-handed G4s, we discovered an opposite foldability pattern compared to right-handed G4s. This inversion is attributed to the reversal of propeller loop geometry when switching helicity, converting long-distance loops into short-distance ones and vice versa. Accordingly, while the +p+p+p topology is the least foldable among right-handed G4s, it shows the highest foldability in left-handed G4s also being the only topology experimentally confirmed for this helicity.^27,28^

Overall, our findings advance the understanding of G-quadruplex conformational space by providing a simple geometric explanation for experimentally observed G4 topological landscape. This comprehensive evaluation of G4 foldability offers valuable insights for the rational design of G-quadruplex structures, which hold significant potential for applications in nanotechnology.^54–56^

## Supplementary Material





## Notes

Visualization of the folding procedure (MP4)
Supporting data and computational methodology for a large-scale MD and machine learning analysis of DNA G-quadruplex folding and topology (PDF)
