# Supplementary material for: Large-Scale Conformational Analysis Explains G‑Quadruplex Topological Landscape
Source: J Phys Chem B. 2025 Sep 11;129(38):9622–32. doi: 10.1021/acs.jpcb.5c04372 (PMC12478849; doi:10.1021/acs.jpcb.5c04372)
Supplement: Supplementary file 2 [file jp5c04372_si_002.pdf]

Supporting Information for: Large-scale Conformational Analysis Explains G-quadruplex Topological Landscape

Michał Jurkowski,† Mateusz Kogut,*,† Michał Olewniczak,† Jan Glinko,‡ and Jacek Czub*,†,¶

†*Department of Physical Chemistry, Gdańsk University of Technology, Narutowicza St 11/12, 80-233 Gdańsk, Poland*

‡*Department of Decision Systems and Robotics, Gdańsk University of Technology, Narutowicza St 11/12, 80-233 Gdańsk, Poland*

¶*BioTechMed Center, Gdańsk University of Technology, Narutowicza St 11/12, 80-233, Gdańsk, Poland*

E-mail: matkogut@pg.edu.pl; jacek.czub@pg.edu.pl

Phone: +48 58 347 27 69; +48 58 347 16 10

SI Methods

Folding procedure – simulation systems and MD protocol

Using the X3DNA web server,[1] we generated initial unfolded structures for 128 DNA oligonucleotides with sequences $G_2T_iG_2T_jG_2T_kG_2$ and $G_3T_iG_3T_jG_3T_kG_3$, where i, j and k ranged from 1 to 4, by creating double-stranded DNA and removing the complementary strand. Each of these 128 single-stranded DNA molecules was placed in a dodecahedral box with a

minimum 1.2 nm distance between any G4 atom and the box edges.

For all MD simulations we used AMBER bsc1 force field.[2] The MD simulations were performed using Gromacs 2020[3] with Plumed 2.6 plugin.[4] Simulations were conducted in the NVT ensemble at 300 K controlled by the v-rescale thermostat.[5] Periodic boundary conditions were applied in 3D, and electrostatic interactions were calculated using the Particle Mesh Ewald (PME)[6] method with a real-space cutoff of 1.2 nm and a Fourier grid spacing of 0.12 nm. A cut-off of 1.2 nm was used for Lennard-Jones potential representing van der Waals interactions. All bond lengths were constrained using P-LINCS.[7] The equations of motion were integrated using the leap-frog algorithm with a 2 fs time step. To accelerate calculations, simulation systems contained only DNA oligonucletide, without water and ions. Instead, we applied implicit solvent with a dielectric constant of 15, chosen to avoid DNA strand collapse at high dielectric constants, while preventing strong phosphate-phosphate repulsion that promotes extended DNA over G4 structures at low dielectric constants. To accelerate calculations, we used implicit solvent with a dielectric constant of 15, chosen to avoid DNA strand collapse at high dielectric constants, while preventing strong phosphate-phosphate repulsion that promotes extended DNA over G4 structures at low dielectric constants.

Preparation of guanine core references

As described in the main text, our folding procedure consisted of four steps, during which a DNA oligonucleotide was folded into the target G4 conformation one G-tract at a time, using four reference structures (unique for each G4 conformation) for the guanine core, each containing one more G-tract than the previous reference.

Here we provide a detailed explanation of how these reference structures were obtained.

For six of the eight possible polarity patterns in three-tetrad G-quadruplexes (LP/LP/LP, LP/RP/LP, RP/LP/LP, RP/LP/RP, RP/RP/LP, RP/RP/RP), we obtained the guanine core references directly from the following experimental structures, respectively (PDB codes): 1kf1,[8] 2mcc,[9] 2jpz,[10] 5j05,[11] 8r4e,[12] and 6r9k.[13] The remaining two references (corresponding

to LP/LP/RP and LP/RP/RP polarity patterns) were assembled from two-tetrad portions of guanine cores extracted from experimental data. Specifically, the LP/LP/RP core was constructed by combining the 5' and middle tetrads from 1kf1 with the middle and 3' tetrads from 5j05, aligning the middle tetrads. Similarly, the LP/RP/RP core was generated by merging the 5' and middle tetrads from 2mcc with the middle and 3' tetrad from 6r9k.

For three of the four possible polarity patterns in two-tetrad G4s (LP/LP, RP/LP, RP/RP), we obtained the guanine core references directly from the following experimental structures, respectively (PDB codes): 2n3m[14] (3'-end G4 block), 2m91,[15] and 7zeo[16] (two G-tetrads formed by continuous 2-nt G-tracts). The remaining LP/RP reference was taken from the two-tetrad portion of a three-tetrad guanine core with LP/RP/LP polarity (PDB code: 2mcc), specifically the 5' and middle tetrads.

For one of the four left-handed two-tetrad G4 polarity patterns (RP/RP), we extracted the guanine core reference from the 5'-terminal G4 block of the experimental structure 4u5m.[17] The remaining three references (LP/LP, LP/RP, RP/LP) were adapted from the corresponding right-handed two-tetrad G4 references by applying a left-handed twist. Specifically, we calculated the right-handed twist angle as the average angle between vectors connecting the diagonally placed N9 atoms in each G-tetrad. We then rotated one of the G-tetrads around the central axis perpendicular to the G-tetrads to create a left-handed twist of equal magnitude.

All guanine core references included only the heavy atoms of the guanine bases (excluding bromine atoms, if they were present in the experimental structure).

After generating guanine core references for every possible polarity pattern, we assigned each guanine in these references to a corresponding guanine in each DNA oligonucleotide considered in this study. This assignment was carried out individually for each topology, ensuring that the guanines' positions within the core references matched their specific locations determined by strand progression and the orientation of G-tracts in the respective topology (see Fig. S3). As a result, we produced a unique set of guanine core references that

unambiguously define both the topology and polarity pattern of G-quadruplexes for all 128 DNA oligonucleotides.

These specially prepared guanine core references were used in the four-step folding procedure (see Fig. S2). Beginning with the 5'-terminal G-tract, each successive step introduced one additional G-tract into the reference, thereby guiding DNA oligonucleotides to fold into desired G4 conformations one G-tract at a time.

Validation of G4 conformations – simulation systems and MD protocols

To asses the range of guanine core fluctuations, we performed unbiased MD simulations initiated from the experimentally determined structures listed in Table S3. Structures were placed in a dodecahedral box, ensuring a minimum distance of 1.2 nm between any G4 atom and the box edges. Each G4 structure was solvated with TIP3P water molecules and appropriate number of K^+ and Cl^- ions was added to neutralize the system and maintain a physiological ionic strength of 150 mM. Additionally, in two- and three-tetrad G4s one and two K^+ ions, respectively, were introduced into the central channel of G4. Simulations were conducted in the NPT ensemble with pressure kept at 1 bar using Parrinello-Rahman barostat.[18] The remaining MD parameters were the same as those used in the folding procedure (see above).

Evaluating G4 foldability determinants via machine learning

To determine the impact of structural features on the foldability of different G4 topologies, we trained an XGBoost classifier to predict whether a set of structural characteristics defining a theoretical G4 conformation leads to a foldable G-quadruplex. Each conformation was described using nine features: the type (−p, +p, −l, +l, d) and length (1–4 nucleotides) of loops at positions I, II, and III, and the polarity (left-polarity LP or right-polarity RP) of

each G-tetrad (T1, T2, T3). The binary target variable for model training was based on our foldability data, with foldable conformations labeled as 1 and unfoldable ones as 0. We used the XGBoost Python package, to train the model.[19]

Subsequently, we calculated Shapley values for all features across the theoretical G4 conformations, using the SHAP python package.[20] Shapley values quantify the contribution of each feature's specific value to the model's prediction. In our case, they indicate how structural features (e.g., loop type +p at position I, loop length of 4 at position II, etc.) affect the prediction of a conformation being foldable or not. Positive Shapley values mean that a feature promotes G4 folding, whereas negative values suggest an unfavorable effect. Finally, to evaluate how each structural feature affects the foldability of different G4 topologies, we averaged the Shapley values over all conformations containing that feature.

Shortest loop path calculation

To find the shortest path a loop must traverse between its attachment points – the C3' and C4' atoms of the guanosines connected by the loop – we calculated the minimal distance between these points along the surface of the guanine core. For this purpose, we first generated a Connolly surface grid for the guanine cores extracted from all properly folded G4 structures obtained through our folding procedure. We employed the Gromacs SASA tool, setting the probe radius to 0.18 nm – the radius of the phosphate group in the DNA backbone – to approximate the loop's radius. As a result, the generated grid points were at distances from the core atoms that are accessible to the loop. The average density of the generated surface grids was $\sim$70 points/nm^2.

Next, we converted the calculated grids into graphs by treating each grid point as a node. Edges were created between nodes that were neighboring points within a 0.3 nm cutoff distance. The edges were assigned weights according to the Euclidean distances between the connected nodes. After constructing the graphs, we used Dijkstra's algorithm to find the shortest paths between the loop attachment points.

Table S1: Summary of all three-tetrad G-quadruplex structures from our de novo folding that have experimentally determined equivalents. 'Top.', 'Seq.', and 'Pol.' stand for G4 topology, lengths of three loops, and G-core polarity patterns, respectively. The RMSD values (in nm) between the folded G4s and experimental references are calculated for G-tetrad stacks (see SI Methods for details). The 'n' column shows the number of matching high-resolution structures deposited in the PDB, with their codes provided in the last column. Structures containing loops of length four or more nucleotides are grouped together.

Top.	Seq.	Pol.	RMSD	n	PDB code
−p−p−p	111	LLL	0.050	8	2le6, 2lee, 2lk7, 2mb2, 2n21, 6ldm, 5dwx, 7oar
	114	LLL	0.048	1	8ebo
	121	LLL	0.057	19	1xav, 2l7v, 2lby, 2lxq, 2lxv, 7n7e, 7n7d, 7kbx, 7kbw, 7kbv, 7e5p 6zl9, 6zl2, 6yy4, 6o2l, 6jj0, 5w77, 5lig, 8dut
		RLL	0.051	1	6erl
	131	LLL	0.054	1	2kze
	141	LLL	0.055	9	2kqg, 2kqh, 2kyp, 2lpw, 2m27, 2m93, 7nwd, 6neb, 6lnz
	212	LLL	0.052	3	5nyu, 5nyt, 5nys
	313	RLL	0.062	4	2l88, 6jwe, 6jwd, 8gp7
	333	LLL	0.081	18	1kf1, 2ld8, 3cdm, 3r6r, 3sc8, 3t5e, 3uyh, 4da3, 4daq, 4fxm, 4g0f 7klp, 6isw, 6ip7, 6ip3, 6h5r, 7pnl, 7qvq
	414	LLL	0.086	1	8edp
+l+p+p	311	RRL	0.051	1	6r9l
		RRR	0.049	1	6r9k
	322	RRL	0.066	1	8r4e
−l−l−p	321	RLL	0.065	1	2kzd
	322	RLL	0.069	1	8r4w
	333	LLL	0.075	1	6ia0
		RLL	0.064	6	2jpz, 2jsl, 2jsq, 6kfj, 6ccw, 5mvb
	341	RLL	0.053	1	2f8u
	421	RLL	0.072	3	7x2z, 7x3a, 8ijc
	431	RLL	0.078	1	8r6d
	432	RLL	0.061	1	186d
	433	RLL	0.065	1	8r6h
−p−l−l	142	RLL	0.056	1	7cv3
	333	LLL	0.053	1	5mbr
		RLL	0.058	11	2e4i, 2gku, 2hy9, 2jsk, 2jsm, 2may, 2mb3, 2mwz, 6kfi, 6ia4, 5z80
+l+p+l	333	RRL	0.057	1	2mbj
−p−p−l	114	LLL	0.068	1	7o1h
+l+l+l	333	RLL	0.065	2	5yey, 8ht7
		RRL	0.055	1	7z9l
−l−l−l	333	RLL	0.051	1	6jkn
		RRL	0.061	1	7otb
	433	RLL	0.046	2	8r6g, 8rw2
+ld−p	441	RLL	0.046	1	6l92
−pd+l	243	LLL	0.061	3	6ffr, 5ov2, 5mcr
		RLL	0.059	2	2lod, 8pse
	443	RLL	0.052	1	8s1w
	444	RLL	0.082	2	5mtg, 5mta
+ld−l	243	RLL	0.063	2	6yep, 6f4z
−ld+l	242	RRL	0.060	1	8psc
	333	LRL	0.081	3	143d, 2mcc, 2mco
	341	RLR	0.068	1	5j05
	342	LRL	0.076	1	6zx7
	343	RLR	0.060	1	6zx6
d+pd	414	RLL	0.052	1	2mft

Table S2: Summary of all two-tetrad G-quadruplex structures from our de novo folding that have experimentally determined equivalents. 'Top.', 'Seq.', and 'Pol.' stand for G4 topology, lengths of three loops, and G-core polarity patterns, respectively. The RMSD values (in nm) between the folded G4s and experimental references are calculated for G-tetrad stacks (see SI Methods for details). The 'n' column shows the number of matching high-resolution structures deposited in the PDB, with their codes provided in the last column. Structures containing loops of length four or more nucleotides are grouped together. Note that any G-quadruplex containing four continuous G-tracts of two guanines (2-nt) is classified here as a "two-tetrad G4", even if an additional G-tetrad arises due to loop guanines, snapback loop or a bulged G-tract.

Top.	Seq.	Pol.	RMSD	n	PDB code
−p−p−p	111	LL	0.056	5	1myq, 1oz8, 2n3m, 8taa, 8xak
	122	LL	0.052	1	6k3y
	124	LL	0.050	1	2m90
	132	LL	0.053	3	7pne, 7png, 8abd
	212	LL	0.053	6	1y8d, 7x7g, 7xdh, 7xh9, 7xhd, 7xie
	221	LL	0.058	1	7wgw
	222	LL	0.053	3	2m4p, 5vhe, 8jfq
	232	LL	0.053	4	2a5p, 2a5r, 2mgn, 2n6c
	234	LL	0.057	5	6t51, 5i2v, 7x8m, 7x8n, 7x8o
	243	LL	0.064	1	2n4y
	321	LL	0.054	1	8psb
	342	LL	0.049	1	7zem
+l+p+p	322	RR	0.059	1	7zeo
	342	RL	0.044	1	7zek
−l−l−p	424	RL	0.058	1	6ac7
	433	RL	0.069	1	2mfu
−l−l−l	232	RL	0.066	2	1hap, 1hut
	324	RL	0.063	1	7w9n
	333	RL	0.050	1	6gh0
+l+l+l	222	RL	0.067	1	8bw5
	232	RL	0.064	27	148d, 1bub, 1c32, 1c34, 1c35, 1c38, 1hao, 1qdf, 1qdh, 1rde, 2lyg 4dih, 4dii, 4lz1, 4lz4, 6z8w, 6z8v, 6gn7, 6evv, 6eo7, 6eo6, 5mjx 5cmx, 7v3t, 7zkl, 7zkm, 7zkn
	242	RL	0.072	3	2m8z, 6fc9, 8abn
	244	RL	0.079	1	7cv4
	444	RL	0.056	9	2km3, 8fhv, 8fhx, 8fhz, 8fi0, 8fi1, 8fi2, 8fi7, 8fi8
+ld−l	343	RL	0.052	1	2kow
	443	RL	0.050	2	5lqh, 8jih
−ld+l	342	RL	0.076	2	2m91, 5j4w
	343	RL	0.044	2	6gzn, 5j4p
	442	RL	0.066	1	2m6v
	443	RL	0.057	5	2kf7, 2kf8, 2kka, 5lqg, 8jic
	444	RL	0.057	1	7oqt
d+pd	424	RL	0.054	1	1i34
+p+p+p	111	RR	0.059	2	2ms9, 4u5m

Table S3: Summary of the G4 structures simulated to characterize the range of structural fluctuations in three types of G-quadruplexes: three-tetrad right-handed, two-tetrad right-handed, and two-tetrad left-handed. Simulation were initiated from selected experimental structures (‘PDB code’). In the table, ‘Top.’ stands for G4 topology, ‘Seq.’ represents the lengths of the three loops, and ‘Pol.’ corresponds to the guanine core polarity patterns. Descriptions of any structural modifications adopted are provided in the ‘Comments’ column.

PDB code	Seq.	Top.	Pol.	Comments
			Right-handed three-tetrad	
2jpz[10]	333	−l−l−p	RLL	5’- and 3’-flanking ends removed
143d[21]	333	−ld+l	LRL	5’-flanking end removed
1kf1[8]	333	−p−p−p	LLL	5’-flanking end removed
			Right-handed two-tetrad	
2mfu[22]	433	−l−l−p	RL	5’- and 3’-flanking ends removed
5j4w[11]	342	−ld+l	RL	–
2n3m[14]	111	−p−p−p	LL	5’-end G4 block and 3’-flanking end removed
			Left-handed two-tetrad	
2ms9[17]	111	+p+p+p	RR	3’-end G4 block and 5’-flanking end removed

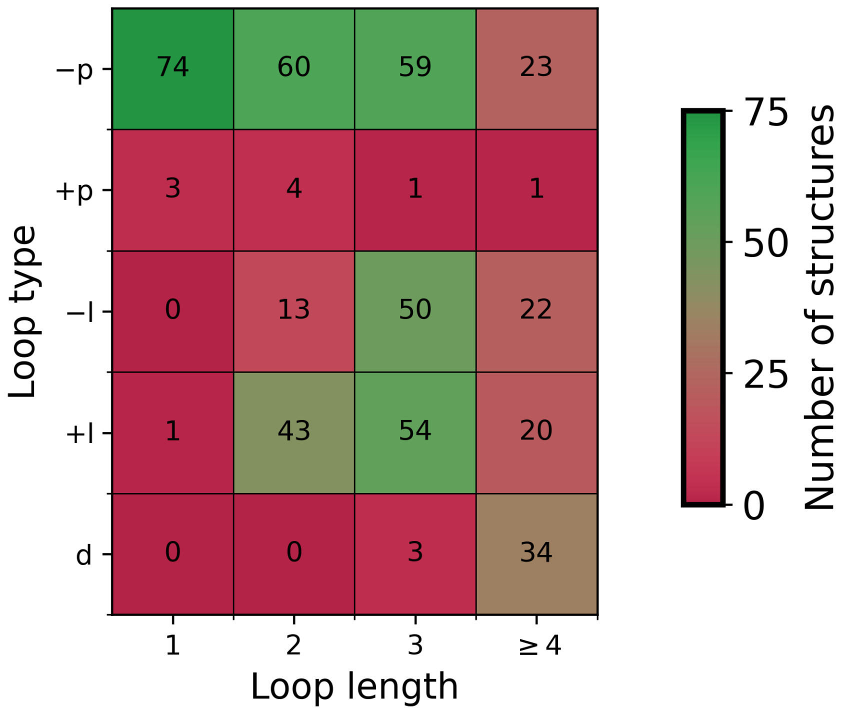

Figure S1: Number of experimentally solved DNA G-quadruplex structures featuring loops of specific types (vertical axis) and lengths (horizontal axis). Loop lengths of four or more nucleotides are grouped together.

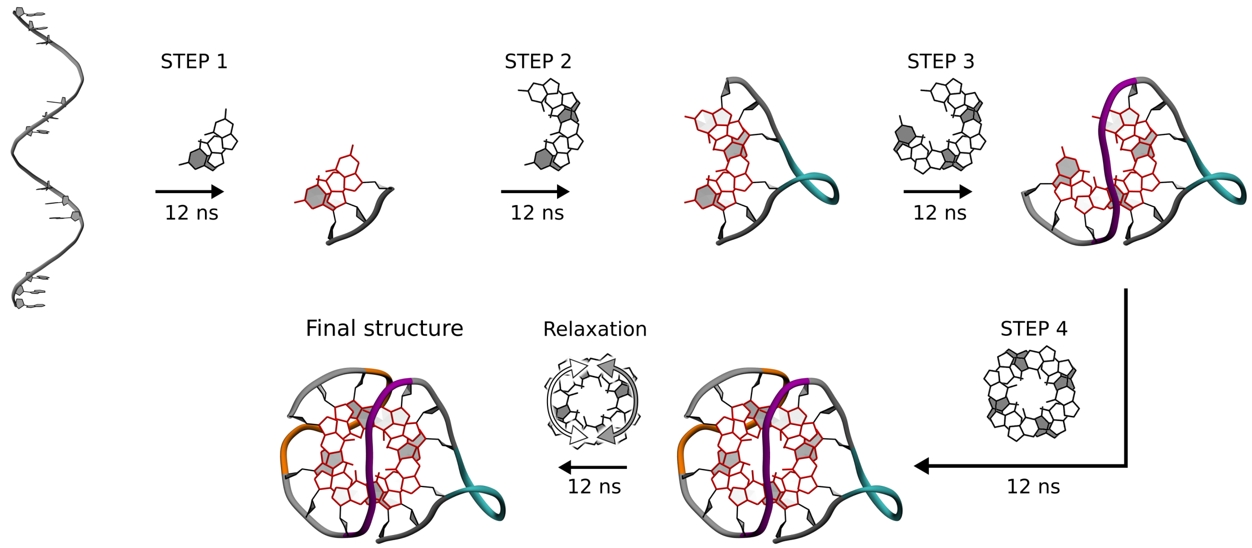

Figure S2: Schematic illustration of the folding procedure, demonstrated using a three-tetrad G4 with the −pd+p topology and RP/LP/LP polarity pattern as an example. In four sequential steps, the DNA oligonucleotide is gradually folded into the target G4 structure using four guanine core references, each containing one more G-tract compared to the previous reference (these references are shown above the arrows). Afterward, a relaxation stage allows the G-tetrads to properly adjust their twist angle (see also Movie S1). For clarity, steps 1–3 omit the DNA segment that has not yet been folded into the reference.

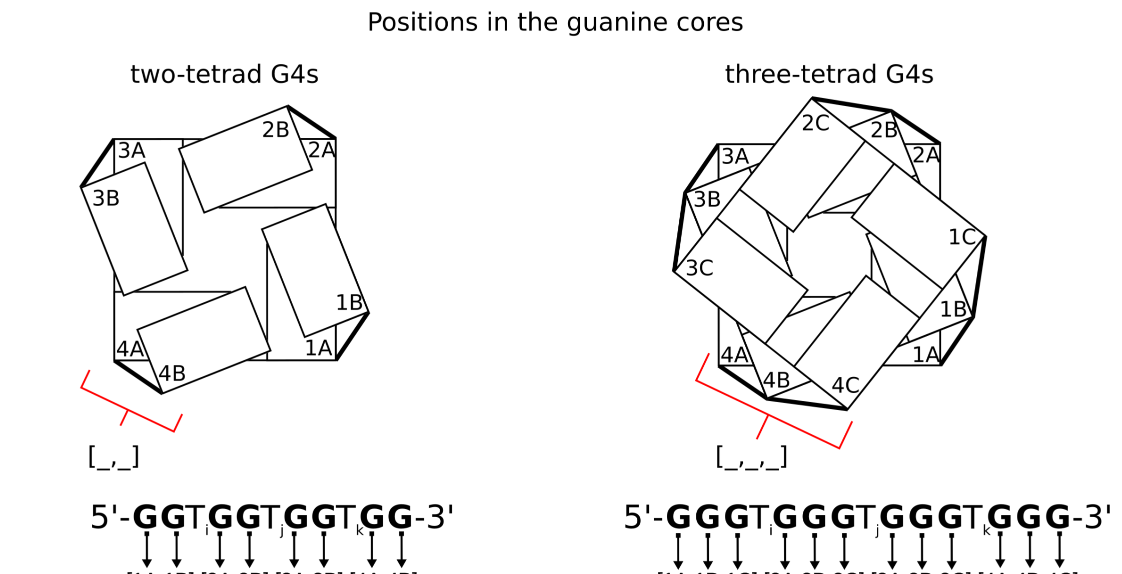

−p−p−p	⟶	[1A,1B] [2A,2B] [3A,3B] [4A,4B]	[1A,1B,1C] [2A,2B,2C] [3A,3B,3C] [4A,4B,4C]
−p−p−l	⟶	[1A,1B] [2A,2B] [3A,3B] [4B,4A]	[1A,1B,1C] [2A,2B,2C] [3A,3B,3C] [4C,4B,4A]
−p−l−l	⟶	[1A,1B] [2A,2B] [3B,3A] [4A,4B]	[1A,1B,1C] [2A,2B,2C] [3C,3B,3A] [4A,4B,4C]
−p−l−p	⟶	[1A,1B] [2A,2B] [3B,3A] [4B,4A]	[1A,1B,1C] [2A,2B,2C] [3C,3B,3A] [4C,4B,4A]
−pd+l	⟶	[1A,1B] [2A,2B] [4B,4A] [3A,3B]	[1A,1B,1C] [2A,2B,2C] [4C,4B,4A] [3A,3B,3C]
−pd+p	⟶	[1A,1B] [2A,2B] [4B,4A] [3B,3A]	[1A,1B,1C] [2A,2B,2C] [4C,4B,4A] [3B,3A,3C]
−l−l−p	⟶	[1A,1B] [2B,2A] [3A,3B] [4A,4B]	[1A,1B,1C] [2C,2B,2A] [3A,3B,3C] [4A,4B,4C]
−l−l−l	⟶	[1A,1B] [2B,2A] [3A,3B] [4B,4A]	[1A,1B,1C] [2C,2B,2A] [3A,3B,3C] [4C,4B,4A]
−l−p−l	⟶	[1A,1B] [2B,2A] [3B,3A] [4A,4B]	[1A,1B,1C] [2C,2B,2A] [3C,3B,3A] [4A,4B,4C]
−l−p−p	⟶	[1A,1B] [2B,2A] [3B,3A] [4B,4A]	[1A,1B,1C] [2C,2B,2A] [3C,3B,3A] [4C,4B,4A]
−ld+p	⟶	[1A,1B] [2B,2A] [4A,4B] [3A,3B]	[1A,1B,1C] [2C,2B,2A] [4A,4B,4C] [3A,3B,3C]
−ld+l	⟶	[1A,1B] [2B,2A] [4A,4B] [3B,3A]	[1A,1B,1C] [2C,2B,2A] [4A,4B,4C] [3C,3B,3A]
d+pd	⟶	[1A,1B] [3B,3A] [2B,2A] [4A,4B]	[1A,1B,1C] [3C,3B,3A] [2C,2B,2A] [4A,4B,4C]
d−pd	⟶	[1A,1B] [3B,3A] [4B,4A] [2A,2B]	[1A,1B,1C] [3C,3B,3A] [4C,4B,4A] [2A,2B,2C]
+pd−l	⟶	[1A,1B] [4A,4B] [2B,2A] [3A,3B]	[1A,1B,1C] [4A,4B,4C] [2C,2B,2A] [3A,3B,3C]
+pd−p	⟶	[1A,1B] [4A,4B] [2B,2A] [3B,3A]	[1A,1B,1C] [4A,4B,4C] [2C,2B,2A] [3C,3B,3A]
+p+p+p	⟶	[1A,1B] [4A,4B] [3A,3B] [2A,2B]	[1A,1B,1C] [4A,4B,4C] [3A,3B,3C] [2A,2B,2C]
+p+p+l	⟶	[1A,1B] [4A,4B] [3A,3B] [2B,2A]	[1A,1B,1C] [4A,4B,4C] [3A,3B,3C] [2C,2B,2A]
+p+l+l	⟶	[1A,1B] [4A,4B] [3B,3A] [2A,2B]	[1A,1B,1C] [4A,4B,4C] [3C,3B,3A] [2A,2B,2C]
+p+l+p	⟶	[1A,1B] [4A,4B] [3B,3A] [2B,2A]	[1A,1B,1C] [4A,4B,4C] [3C,3B,3A] [2C,2B,2A]
+ld−p	⟶	[1A,1B] [4B,4A] [2A,2B] [3A,3B]	[1A,1B,1C] [4C,4B,4A] [2A,2B,2C] [3A,3B,3C]
+ld−l	⟶	[1A,1B] [4B,4A] [2A,2B] [3B,3A]	[1A,1B,1C] [4C,4B,4A] [2A,2B,2C] [3C,3B,3A]
+l+l+p	⟶	[1A,1B] [4B,4A] [3A,3B] [2A,2B]	[1A,1B,1C] [4C,4B,4A] [3A,3B,3C] [2A,2B,2C]
+l+l+l	⟶	[1A,1B] [4B,4A] [3A,3B] [2B,2A]	[1A,1B,1C] [4C,4B,4A] [3A,3B,3C] [2C,2B,2A]
+l+p+l	⟶	[1A,1B] [4B,4A] [3B,3A] [2A,2B]	[1A,1B,1C] [4C,4B,4A] [3C,3B,3A] [2A,2B,2C]
+l+p+p	⟶	[1A,1B] [4B,4A] [3B,3A] [2B,2A]	[1A,1B,1C] [4C,4B,4A] [3C,3B,3A] [2C,2B,2A]

Figure S3: To generate a set of guanine core references that define each topology and polarity pattern for all 128 DNA oligonucleotides, we mapped each guanine in the reference to a corresponding guanine in the DNA oligonucleotide using the coding scheme shown. In this scheme, digits (1–4) denote the G-tract positions within the guanine core, and letters (A, B for two-tetrad G4s and A, B, C for three-tetrad G4s) indicate the G-tetrads. A unique mapping for guanines in the oligonucleotides to their respective positions in the guanine core reference is illustrated for all 26 topologies. Codes for guanines belonging to the same G-tract appear in brackets for clarity.

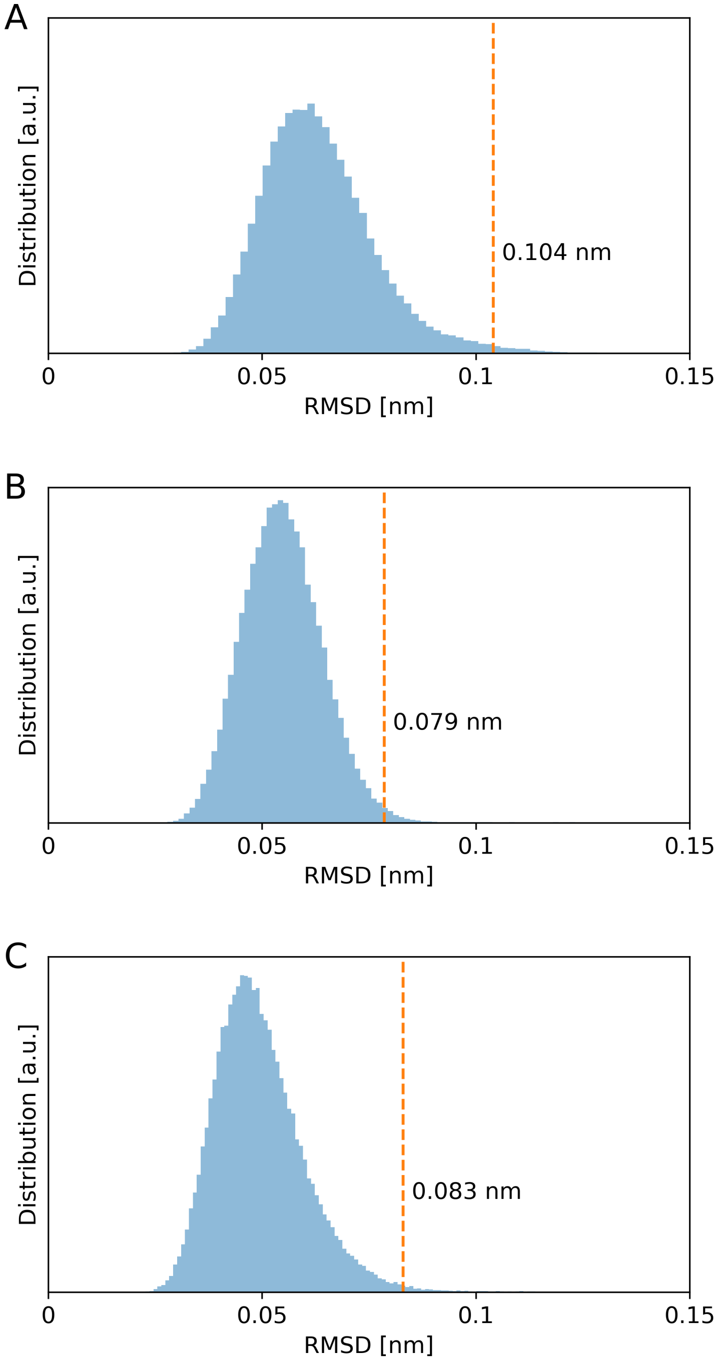

Figure S4: Distributions of RMSD values calculated for the guanine core relative to reference structures, characterizing the range of structural fluctuations in three types of G-quadruplexes: (**A**) three-tetrad right-handed G-quadruplexes, calculated from three independent 1μs MD simulations initiated from experimental structures 2jpz, 143d, and 1kf1; (**B**) two-tetrad right-handed G-quadruplexes, calculated from three independent 1 μs MD simulations initiated from experimental structures 2mfu, 5j4w, and 2n3m; (**C**) two-tetrad left-handed G-quadruplexes, calculated from one 3 μs MD simulation initiated from experimental structure 2ms9. Vertical dashed lines represent the 99th percentile of each distribution.

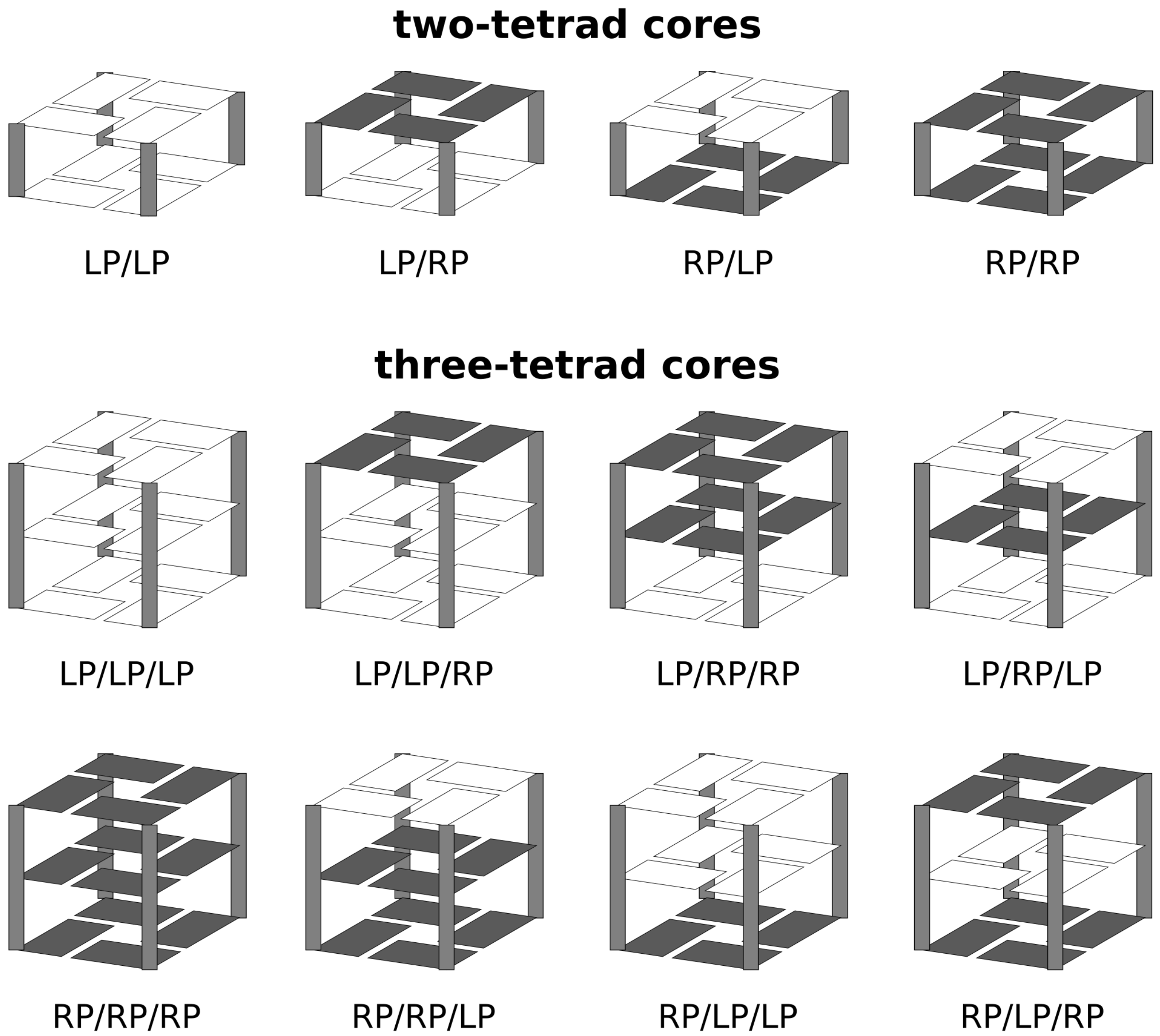

Figure S5: Schematic representations of all possible guanine core polarity patterns for two- and three-tetrad G-quadruplexes. White and gray G-tetrads correspond to left polarity (LP) and right polarity (RP), respectively.

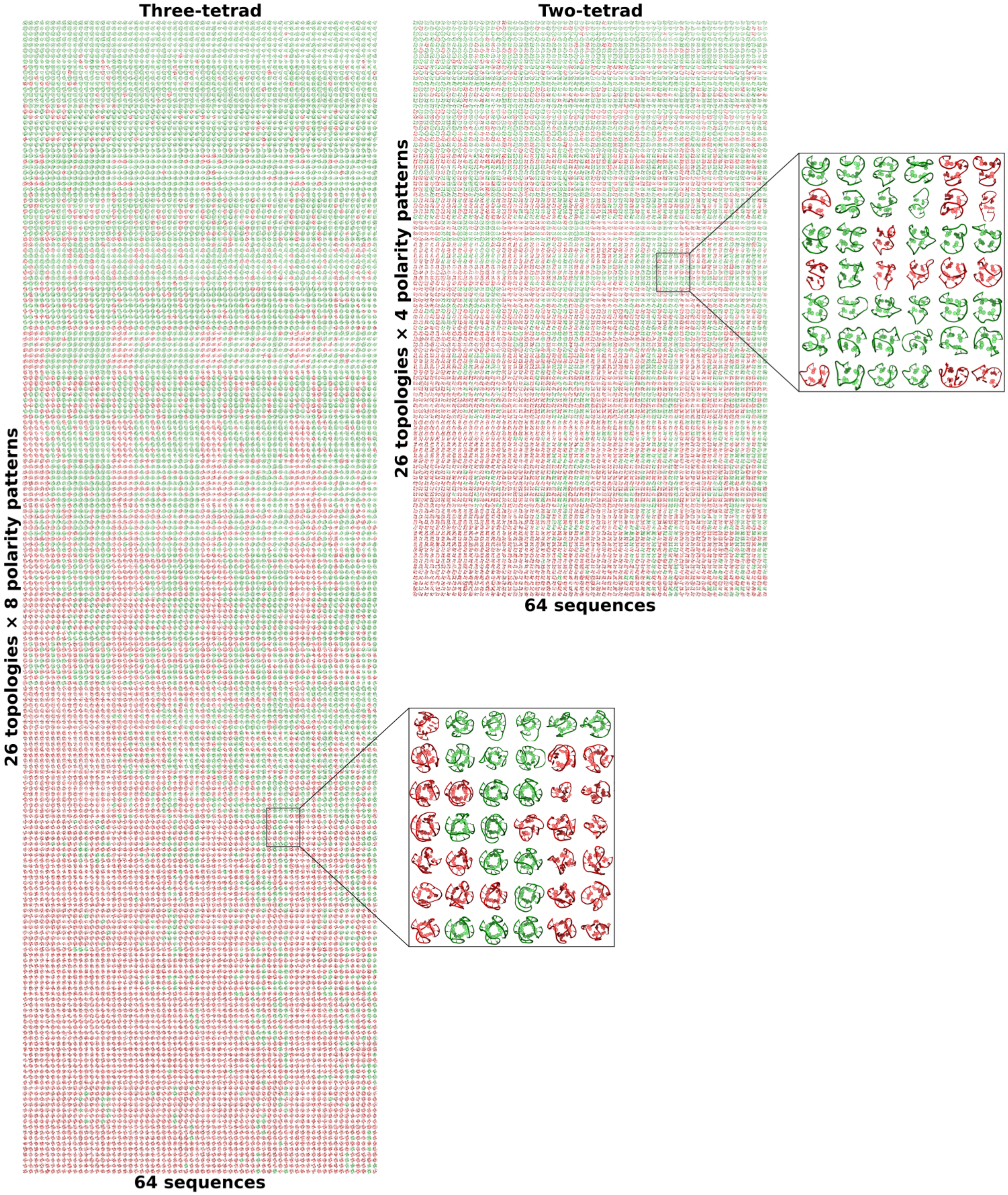

Figure S6: Shown are all of the structures obtained by our folding procedure. Structures with RMSD values below the threshold (see Methods) are shown in green, whereas those exceeding the threshold appear in red. All structures and their high-resolution visualizations are available at DOI: 10.34808/fcyz-w866.

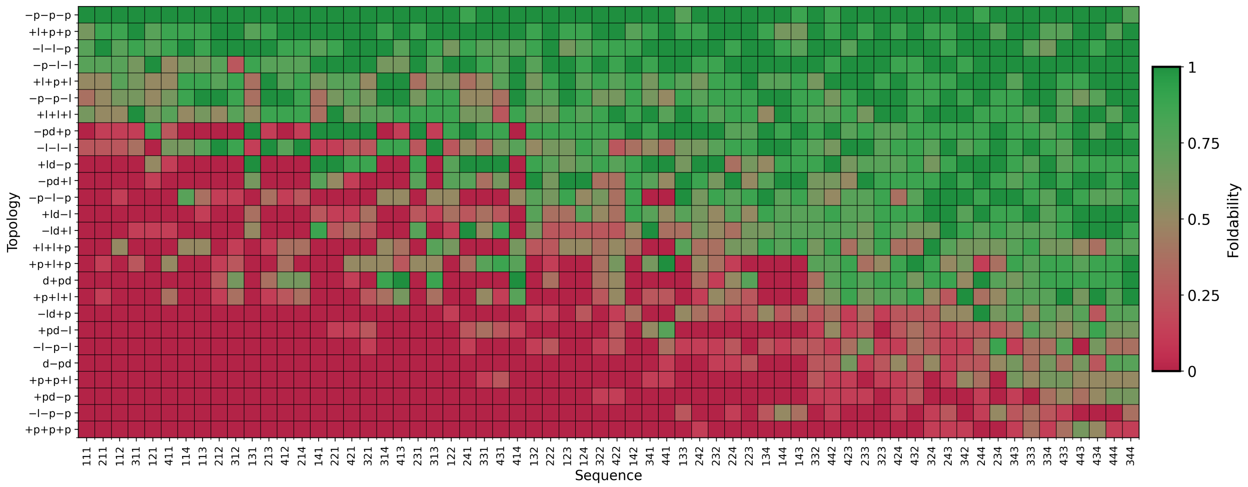

Figure S7: Foldability for each of the 26 topologies across all 64 considered sequences capable of forming three-tetrad G4s, calculated over eight possible polarity patterns. Each sequence is denoted by a three-digit code indicating the lengths of its three loop regions.

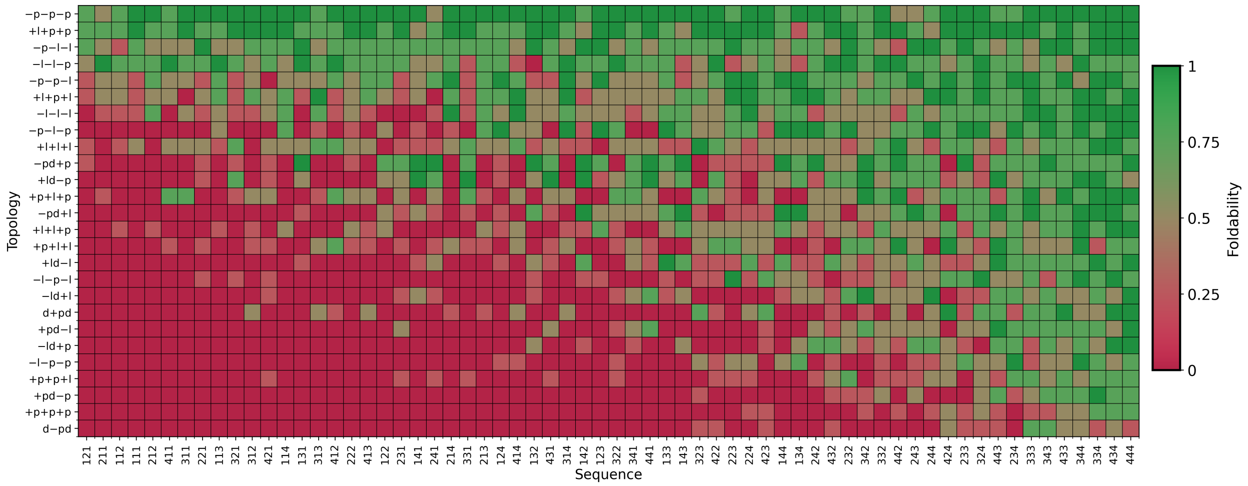

Figure S8: Foldability for each of the 26 topologies across all 64 considered sequences capable of forming two-tetrad G4s, calculated over eight possible polarity patterns. Each sequence is denoted by a three-digit code indicating the lengths of its three loop regions.

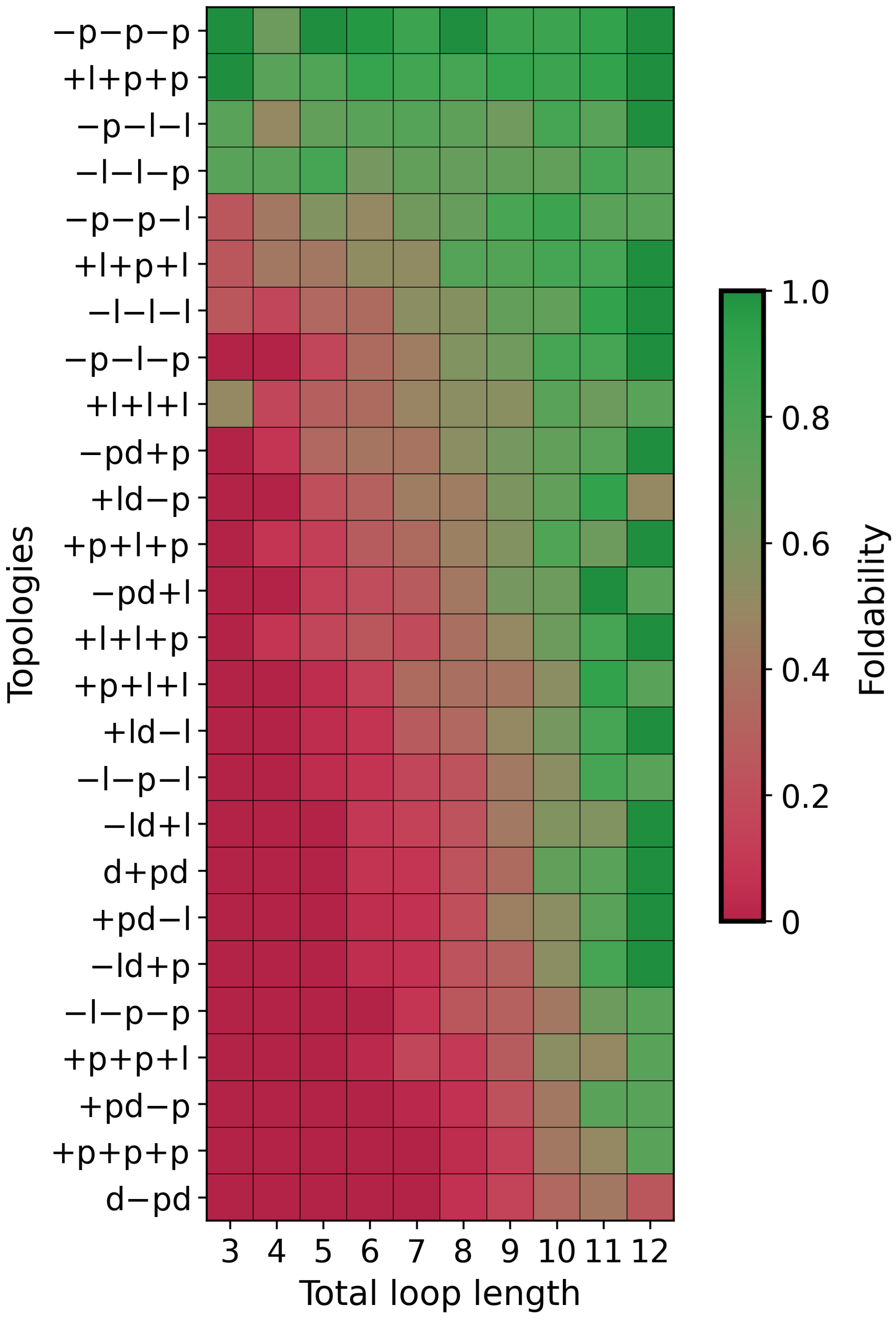

Figure S9: Foldability for each of the 26 topologies of right-handed two-tetrad G-quadruplexes, calculated as the fraction of G4-foldable structures among all sequences with the same total loop length (x-axis) across all eight polarity patterns. The topologies (y-axis) are arranged in order of decreasing average foldability.

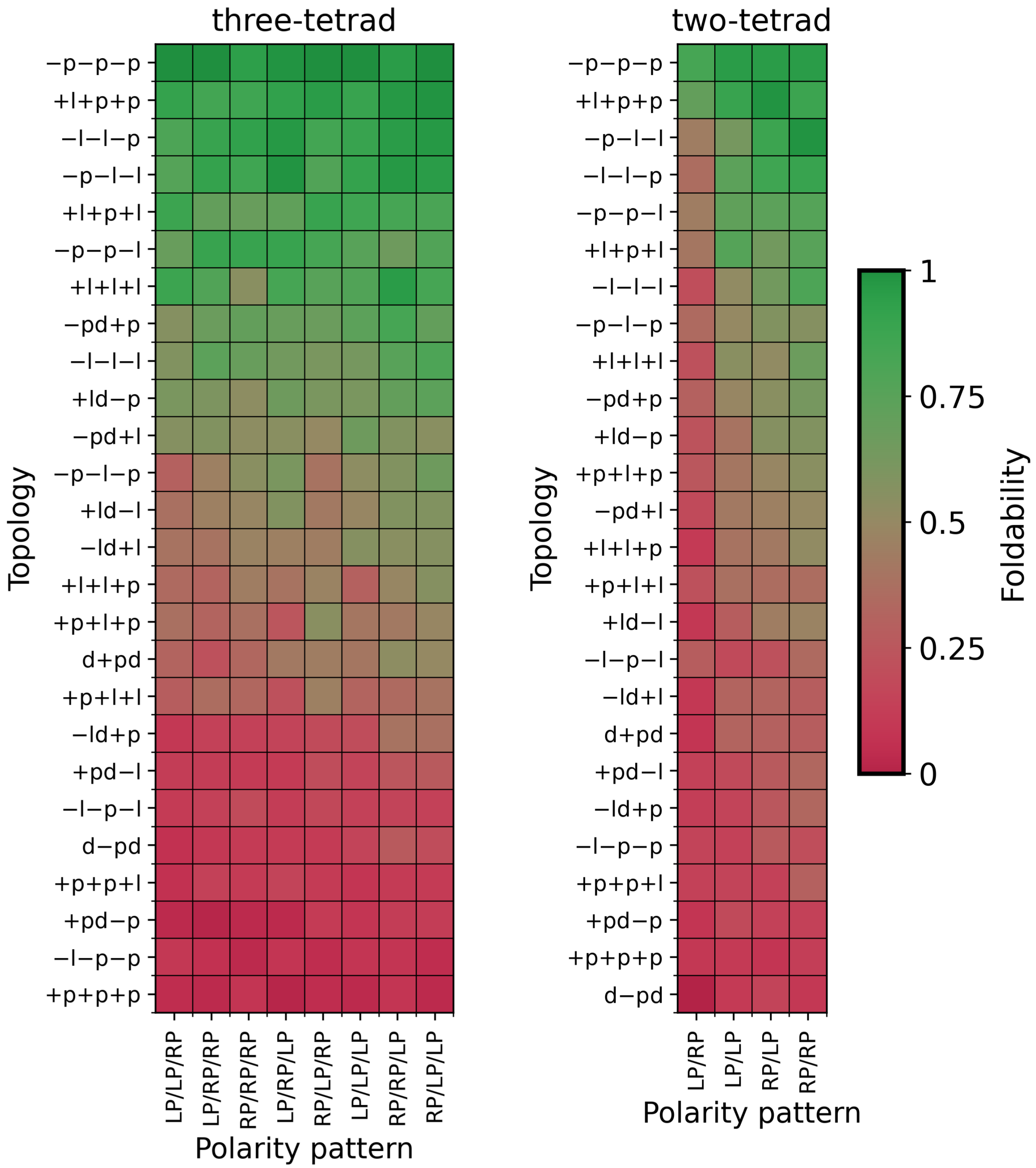

Figure S10: Foldability for each of the 26 topologies of right-handed three-tetrad (left) and right-handed two-tetrad (right) G-quadruplexes across all possible G-core polarity patterns, calculated over all 64 sequences considered.

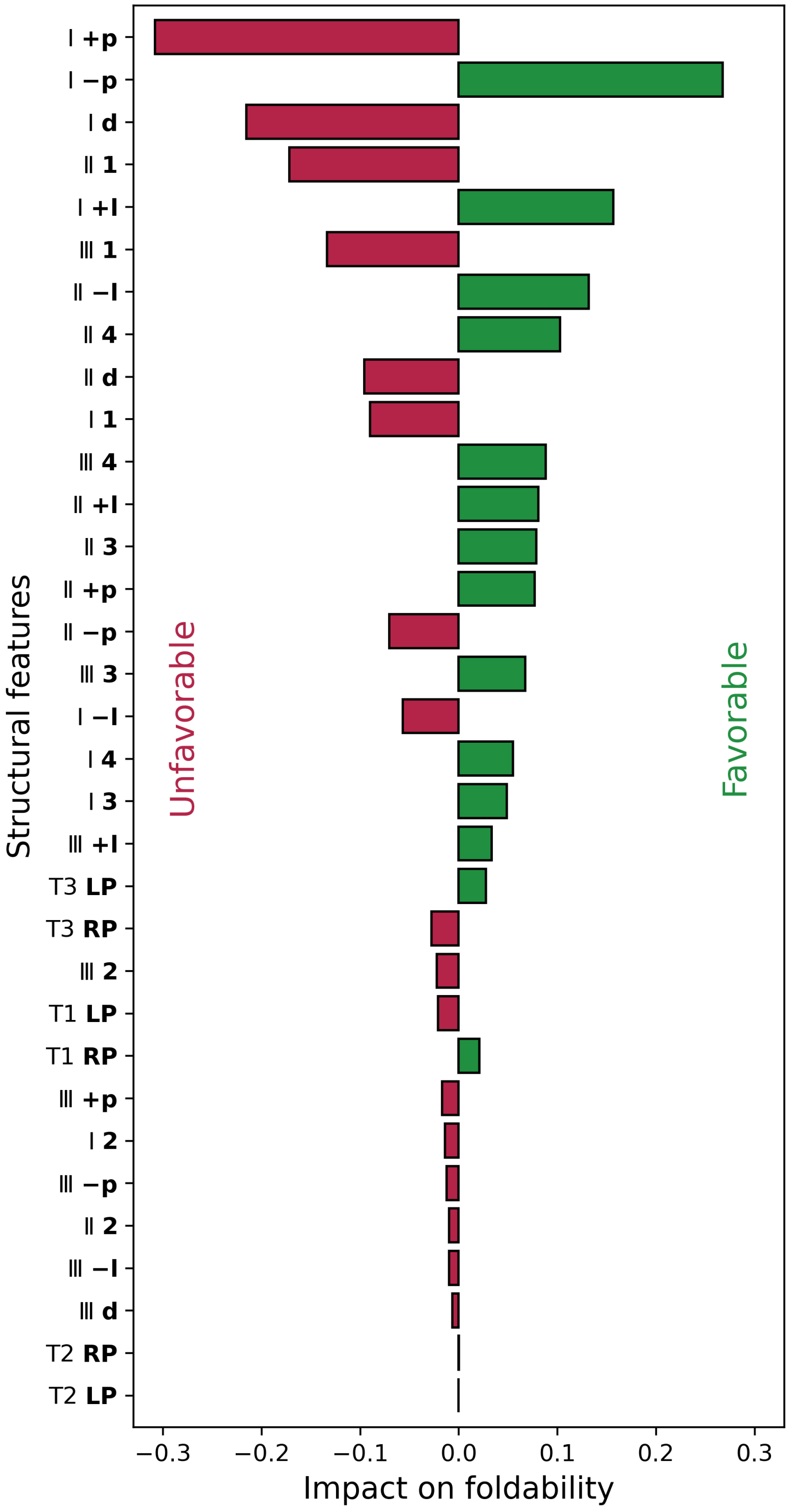

Figure S11: Impact of all considered structural features on foldability of right-handed three-tetrad G4s, as determined by their average Shapley values in the XGBoost foldability prediction model. Feature labels indicate the loop position (Roman numerals) and the loop characteristic (length or type in bold).

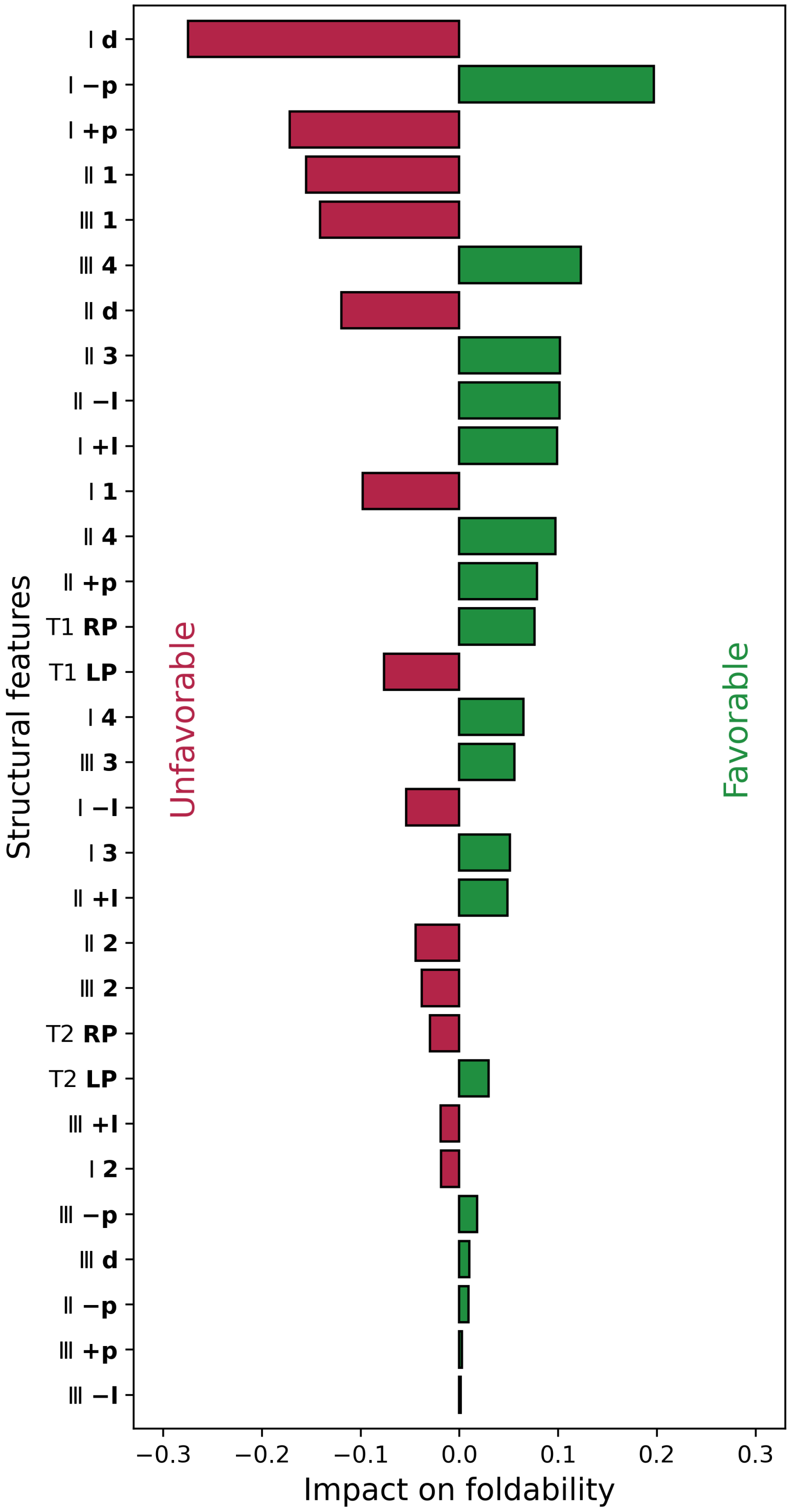

Figure S12: Impact of all considered structural features on foldability of right-handed two-tetrad G4s, as determined by their average Shapley values in the XGBoost foldability prediction model. Feature labels indicate the loop position (Roman numerals) and the loop characteristic (length or type in bold).

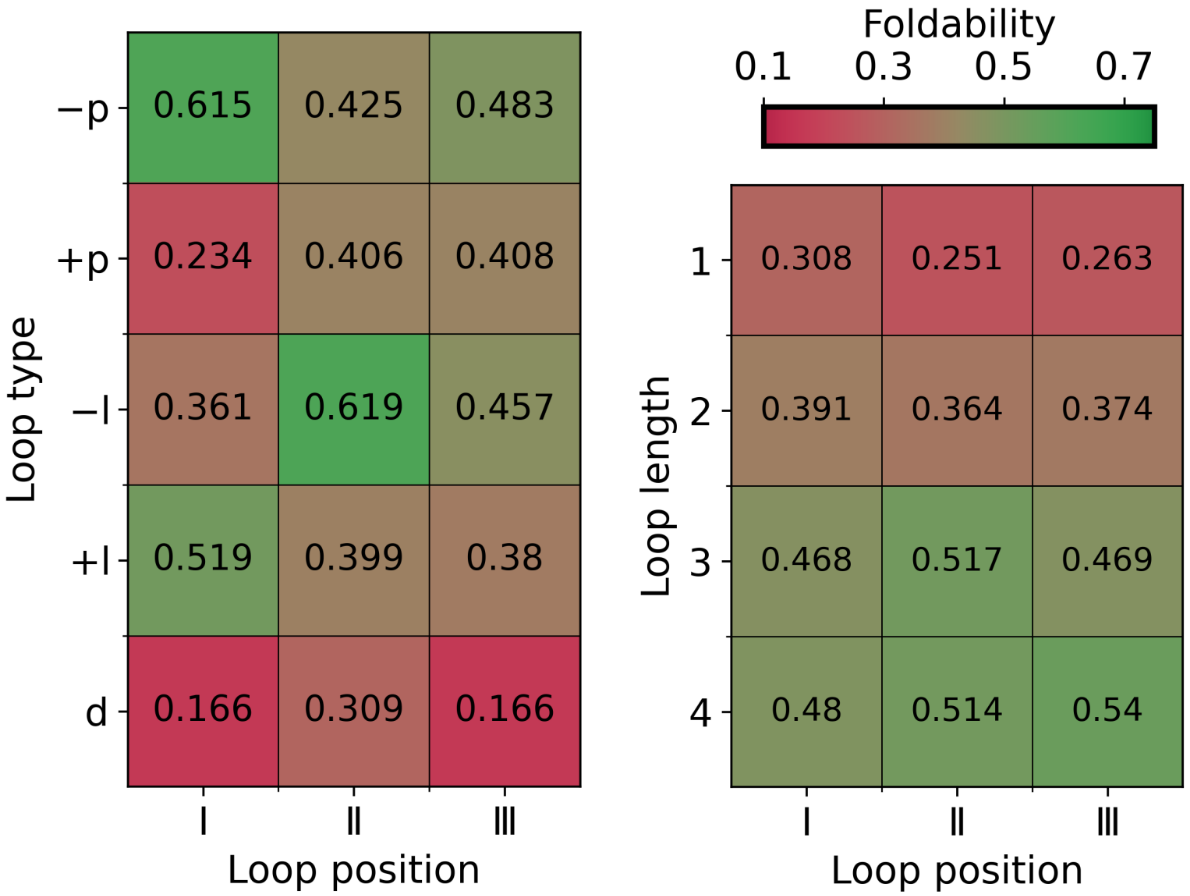

Figure S13: Average foldability of G4 topologies with specific loop types (left) and loop lengths (right) at each of three positions, calculated for two-tetrad G-quadruplexes.

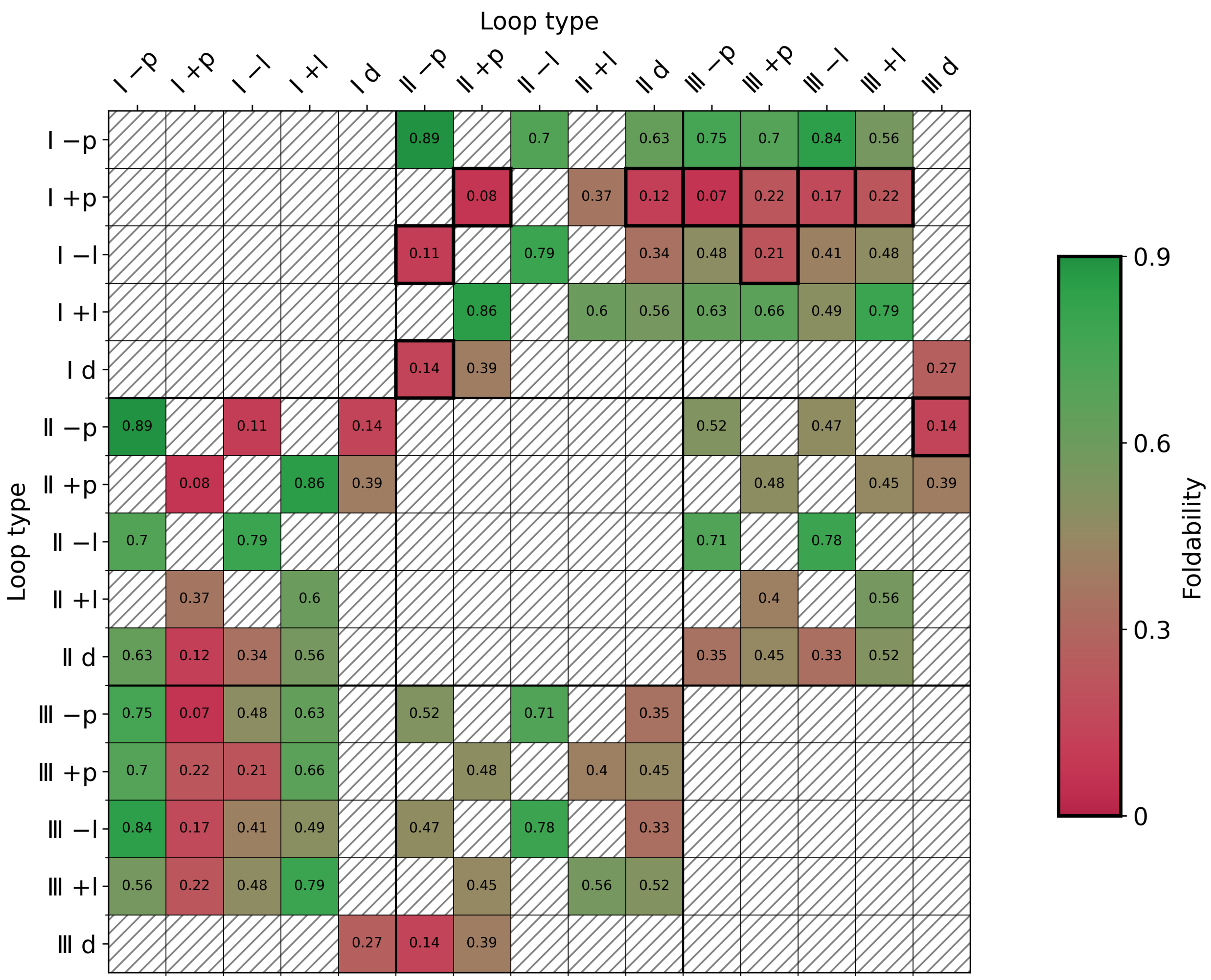

Figure S14: Average foldability of topologies featuring all possible combinations of loop types at any of two positions (two-loop combinations), calculated for three-tetrad G-quadruplexes. Squares corresponding to two-loop combinations for which foldability is 0.25 or lower are outlined with thicker frames. Prohibited two-loop combinations are indicated by hatched squares.

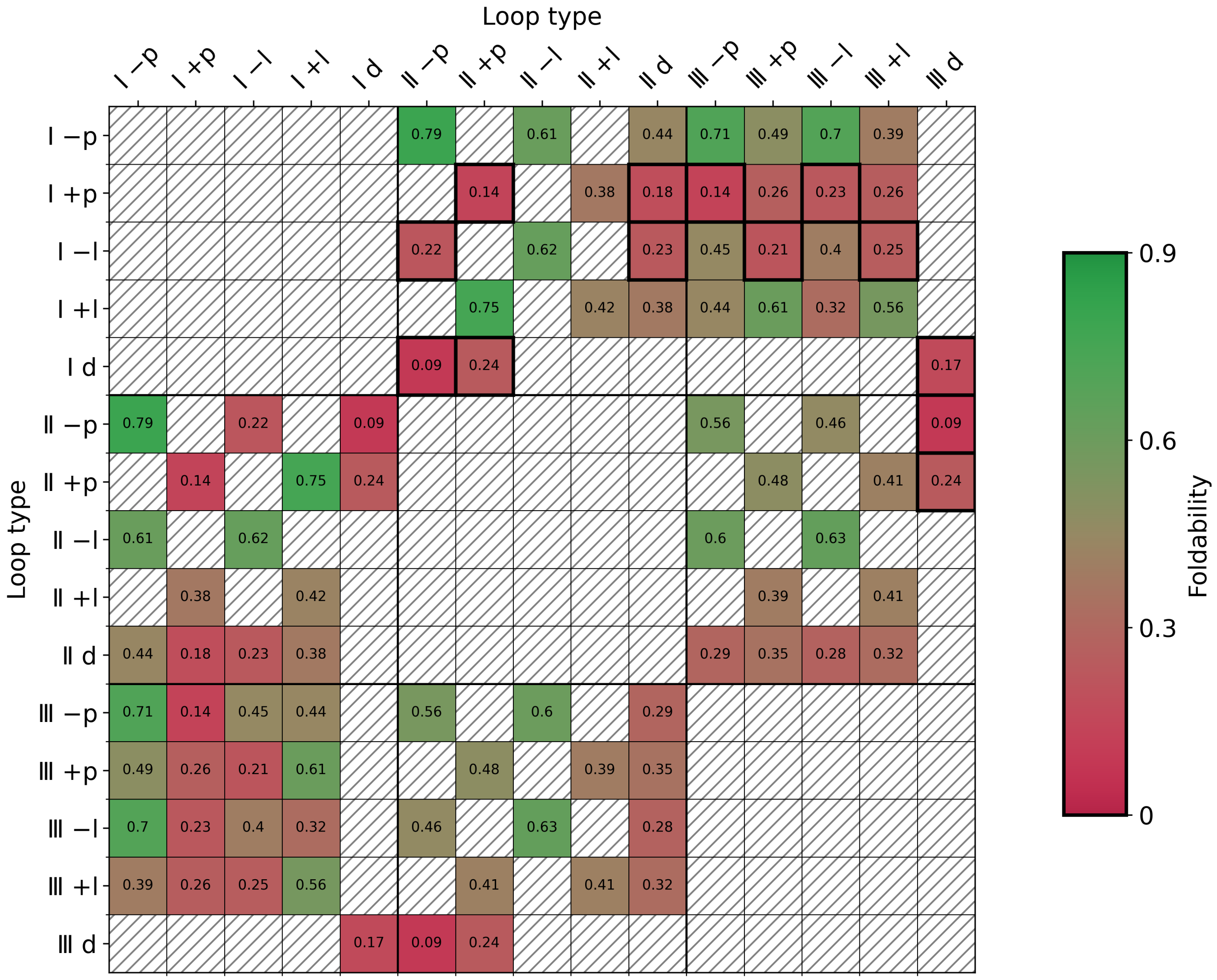

Figure S15: Average foldability of topologies featuring all possible combinations of loop types at any of two positions (two-loop combinations), calculated for two-tetrad G-quadruplexes. Squares corresponding to two-loop combinations for which foldability is 0.25 or lower are outlined with thicker frames. Prohibited two-loop combinations are indicated by hatched squares.

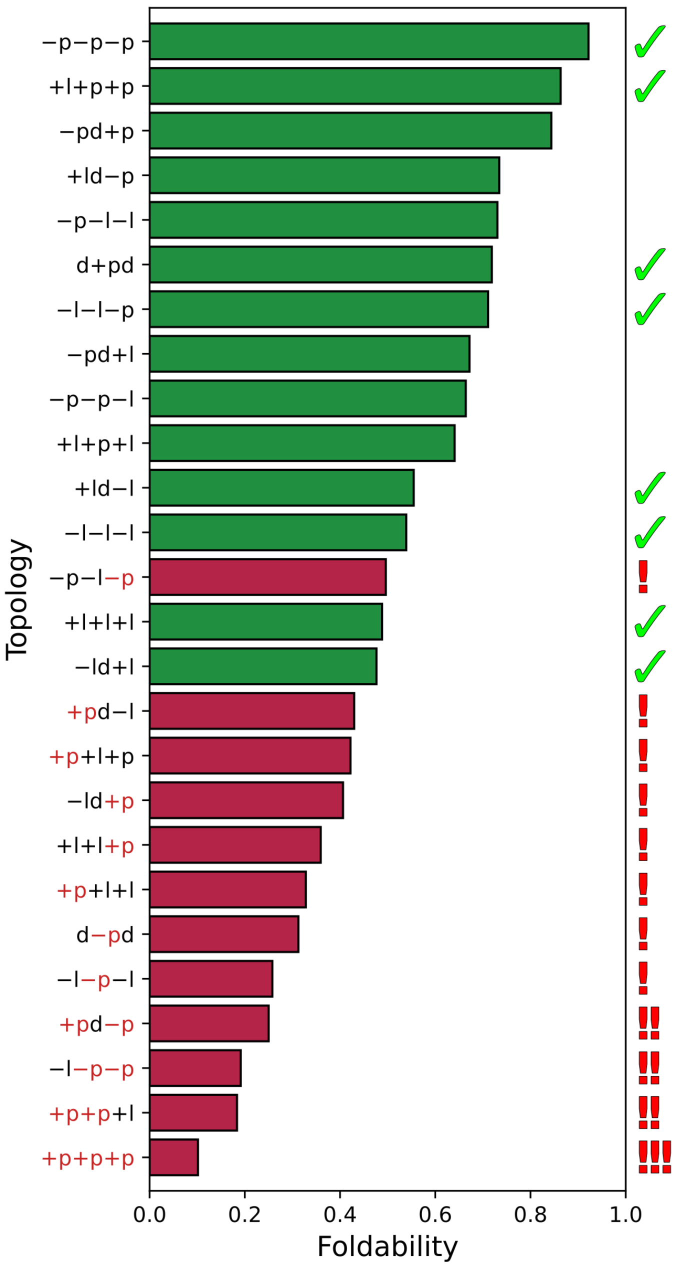

Figure S16: Foldability of each of the 26 right-handed two-tetrad G-quadruplex topologies, calculated across all folded structures while excluding those with diagonal loops shorter than three nucleotides since they are not foldable. Topologies that include long-distance propeller loops are shown in red, with exclamation marks on the right indicating the number of such loops in each topology. Topologies without long-distance propellers are shown in green. Topologies confirmed by experimental right-handed G4 structures are marked with ticks.

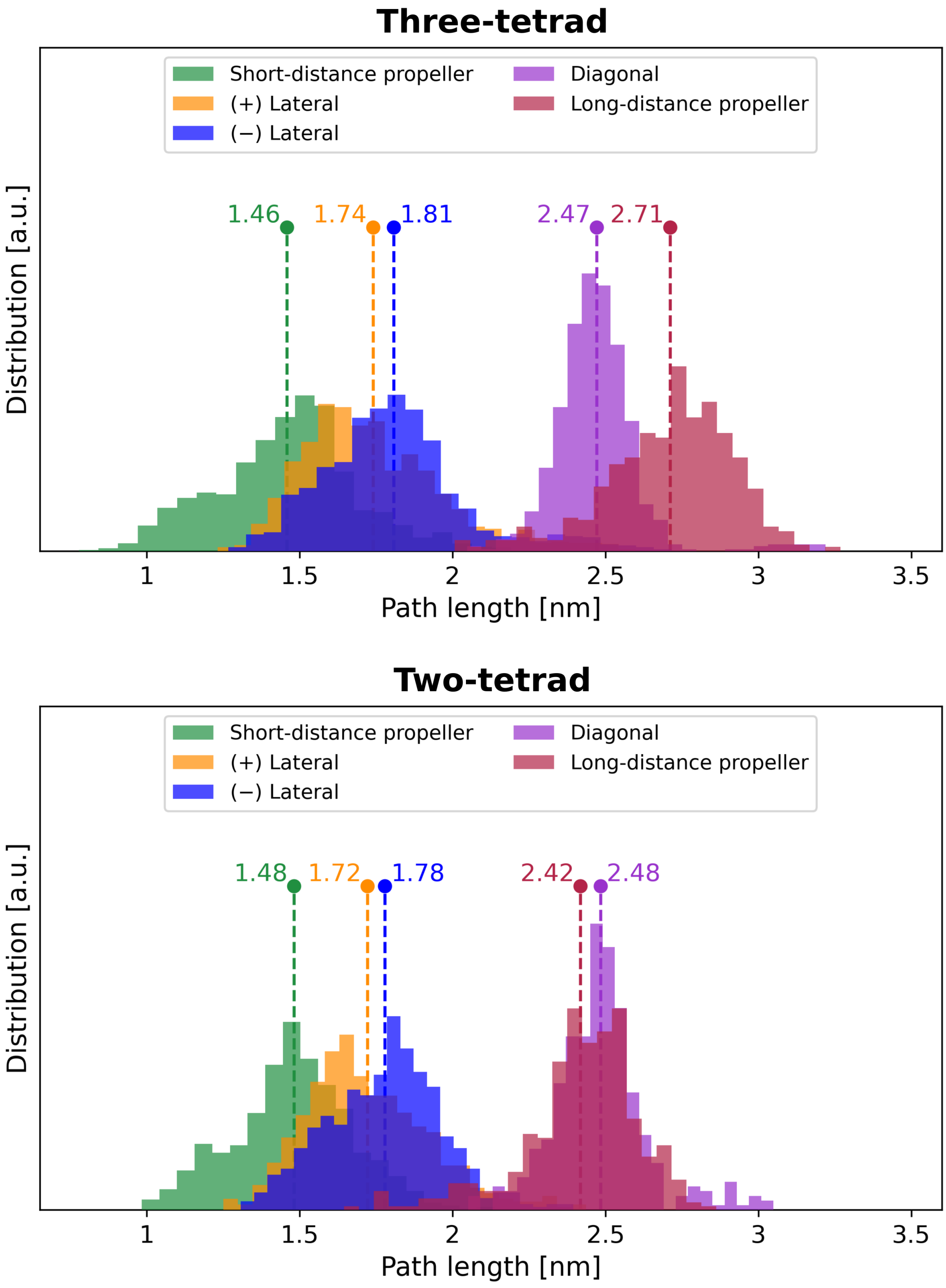

Figure S17: Distribution of the lengths of the shortest paths between loops' attachment points (see SI Methods), calculated for short-distance propeller loops, (+) lateral loops, (−) lateral loops, diagonal loops and long-distance propeller loops, across all foldable three-tetrad (top) and two-tetrad (bottom) G4s. Vertical dashed lines indicate the average path length for each distribution.

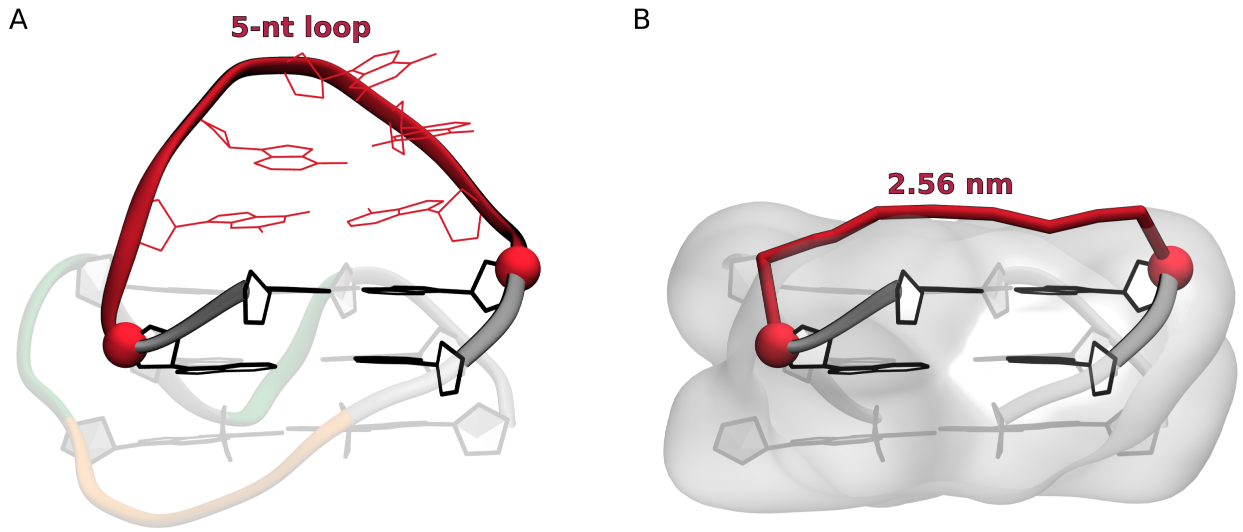

Figure S18: (**A**) Experimental structure of a G-quadruplex containing a snapback long-distance propeller loop highlighted in red (PDB code: 2O3M[23]). The loop's attachment points (C3' and C4' atoms) are shown as red spheres. (**B**) Connolly surface of the G-core extracted from the 2O3M structure, with the shortest path between the attachment points of the long-distance propeller loop depicted in red stick representation.

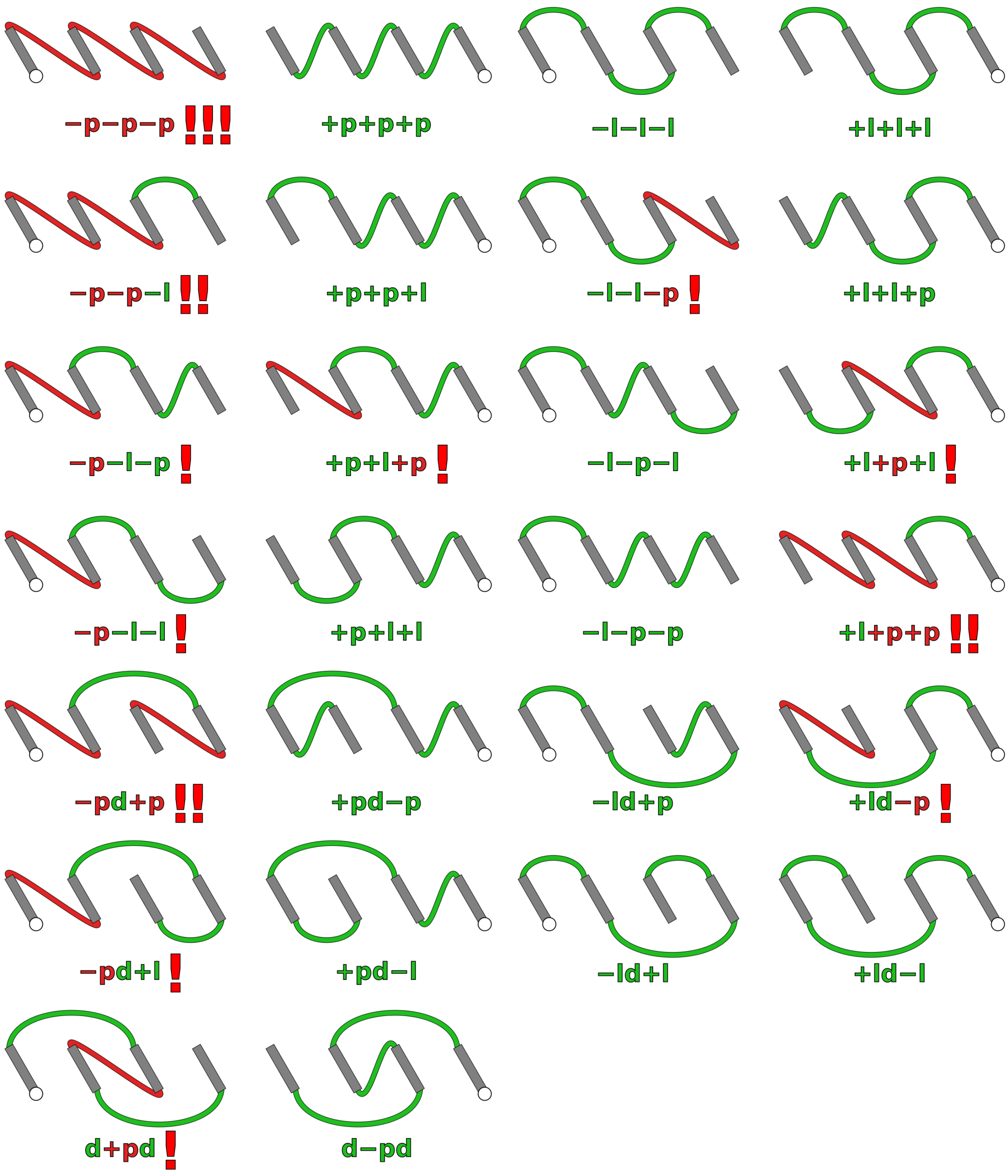

Figure S19: Two-dimensional projections of all 26 G4 topologies with left-handed helicity. Long-distance propeller loops appear in red, while the other loop types are in green. Exclamation marks indicate the number of long-distance propellers within each topology. 5'-ends are marked with white circles.

Movie S1: Visualization of the folding procedure for 3 different G4 topologies: −pd+p (left), −p−p−p (middle) and +l+l+l (right) with RP/LP/LP, LP/LP/LP and LP/RP/RP polarity patterns, respectively. For all 3 conformations, folding was initiated from oligonucleotide with $G_3T_3G_3T_3G_3T_3G_3$ sequence in unfolded state. G-tracts are presented in gray while propeller, lateral and diagonal loops in cyan, orange and magenta, respectively, consistently with Fig. 1. Sugar and base atoms of thymines are hidden for clarity, reappearing at the end of the movie when G4 structures are fully formed. Simulation time is given in the bottom right corner. The movie was prepared using VMD[24] and the Molywood package.[25]

References

(1) Lu, X.-J.; Olson, W. K. 3DNA: a software package for the analysis, rebuilding and visualization of three-dimensional nucleic acid structures. *Nucleic Acids Res.* **2003**, *31*, 5108–5121.

(2) Ivani, I.; Dans, P. D.; Noy, A.; Pérez, A.; Faustino, I.; Hospital, A.; Walther, J.; Andrio, P.; Goñi, R.; Balaceanu, A.; others Parmbsc1: a refined force field for DNA simulations. *Nat. Methods* **2016**, *13*, 55–58.

(3) Abraham, M. J.; Murtola, T.; Schulz, R.; Páll, S.; Smith, J. C.; Hess, B.; Lindahl, E. GROMACS: High performance molecular simulations through multi-level parallelism from laptops to supercomputers. *SoftwareX* **2015**, *1*, 19–25.

(4) Tribello, G. A.; Bonomi, M.; Branduardi, D.; Camilloni, C.; Bussi, G. PLUMED 2: New feathers for an old bird. *Comput. Phys. Commun.* **2014**, *185*, 604–613.

(5) Bussi, G.; Donadio, D.; Parrinello, M. Canonical sampling through velocity rescaling. *J. Chem. Phys.* **2007**, *126*.

(6) Darden, T.; York, D.; Pedersen, L. Particle mesh Ewald: An N·log (N) method for Ewald sums in large systems. *J. Chem. Phys.* **1993**, *98*, 10089–10092.

(7) Hess, B. P-LINCS: A parallel linear constraint solver for molecular simulation. *J. Chem. Theory Comput.* **2008**, *4*, 116–122.

(8) Parkinson, G. N.; Lee, M. P.; Neidle, S. Crystal structure of parallel quadruplexes from human telomeric DNA. *Nature* **2002**, *417*, 876–880.

(9) Wilson, T.; Costa, P. J.; Feélix, V.; Williamson, M. P.; Thomas, J. A. Structural studies on dinuclear ruthenium (II) complexes that bind diastereoselectively to an antiparallel folded human telomere sequence. *J. Med. Chem.* **2013**, *56*, 8674–8683.

(10) Dai, J.; Carver, M.; Punchihewa, C.; Jones, R. A.; Yang, D. Structure of the Hybrid-2 type intramolecular human telomeric G-quadruplex in K+ solution: insights into structure polymorphism of the human telomeric sequence. *Nucleic Acids Res.* **2007**, *35*, 4927–4940.

(11) Dvorkin, S. A.; Karsisiotis, A. I.; Webba da Silva, M. Encoding canonical DNA quadruplex structure. *Sci. Adv.* **2018**, *4*, eaat3007.

(12) Jana, J.; Vianney, Y. M.; Weisz, K. Impact of loop length and duplex extensions on the design of hybrid-type G-quadruplexes. *Chem. Commun.* **2024**, *60*, 854–857.

(13) Karg, B.; Mohr, S.; Weisz, K. Duplex-guided refolding into novel G-quadruplex (3+ 1) hybrid conformations. *Angew. Chem. Int. Ed.* **2019**, *58*, 11068–11071.

(14) Do, N. Q.; Chung, W. J.; Truong, T. H. A.; Heddi, B.; Phan, A. T. G-quadruplex structure of an anti-proliferative DNA sequence. *Nucleic Acids Res.* **2017**, *45*, 7487–7493.

(15) Lim, K. W.; Phan, A. T. Structural basis of DNA quadruplex-duplex junction formation. *Angew. Chem* **2013**, *52*, 8566–8569.

(16) Jana, J.; Vianney, Y. M.; Schröder, N.; Weisz, K. Guiding the folding of G-quadruplexes through loop residue interactions. *Nucleic Acids Res.* **2022**, *50*, 7161–7175.

(17) Chung, W. J.; Heddi, B.; Schmitt, E.; Lim, K. W.; Mechulam, Y.; Phan, A. T. Structure of a left-handed DNA G-quadruplex. *Proc. Natl. Acad. Sci. USA* **2015**, *112*, 2729–2733.

(18) Parrinello, M.; Rahman, A. Polymorphic transitions in single crystals: A new molecular dynamics method. *J. Appl. Phys.* **1981**, *52*, 7182–7190.

(19) Chen, T.; Guestrin, C. XGBoost: A Scalable Tree Boosting System. Proceedings of the 22nd ACM SIGKDD International Conference on Knowledge Discovery and Data Mining. New York, NY, USA, 2016; pp 785–794.

(20) Lundberg, S. M.; Erion, G.; Chen, H.; DeGrave, A.; Prutkin, J. M.; Nair, B.; Katz, R.; Himmelfarb, J.; Bansal, N.; Lee, S.-I. From local explanations to global understanding with explainable AI for trees. *Nat. Mach. Intell.* **2020**, *2*, 2522–5839.

(21) Wang, Y.; Patel, D. J. Solution structure of the human telomeric repeat d [AG3 (T2AG3) 3] G-tetraplex. *Structure* **1993**, *1*, 263–282.

(22) Karsisiotis, A. I.; Dillon, P.; Webba da Silva, M. Solution NMR structure of quadruplex d(TGGGTTTGGGTTGGGTTTGGG) in sodium conditions. 2013; https://doi.org/10.2210/pdb2mfu/pdb.

(23) Phan, A. T.; Kuryavyi, V.; Burge, S.; Neidle, S.; Patel, D. J. Structure of an unprecedented G-quadruplex scaffold in the human c-kit promoter. *J. Am. Chem. Soc.* **2007**, *129*, 4386–4392.

(24) Humphrey, W.; Dalke, A.; Schulten, K. VMD: visual molecular dynamics. *J. Mol. Graphics* **1996**, *14*, 33–38.

(25) Wieczór, M.; Hospital, A.; Bayarri, G.; Czub, J.; Orozco, M. Molywood: streamlining the design and rendering of molecular movies. *Bioinformatics* **2020**, *36*, 4660–4661.
